# The prognostic value of the lung immune prognostic index in patients with urological cancers: a systematic review and meta-analysis

**DOI:** 10.3389/fimmu.2026.1806105

**Published:** 2026-03-25

**Authors:** Zhaojie Lyu, Yuhan Xiao, Jie Wang, Ruicheng Wu, Koo Han Yoo, Wuran Wei, Qi Zhang, Yandong Xie, Dechao Feng

**Affiliations:** 1 Department of Urology, Shenzhen Key Laboratory of Male Reproduction and Genetics, Institute of Precision Medicine, Peking University Shenzhen Hospital, Shenzhen, Guangdong, China; 2 Department of Urology, Institute of Urology, West China Hospital, Sichuan University, Chengdu, China; 3 Division of Surgery and Interventional Science, University College London, London, United Kingdom; 4 Department of Urology, Kyung Hee University, Seoul, Republic of Korea; 5 Urology and Nephrology Center, Department of Urology, Zhejiang Provincial People’s Hospital (Affiliated People’s Hospital), Hangzhou Medical College, Hangzhou, Zhejiang, China; 6 Department of Urology, Nanfang Hospital, Southern Medical University, Guangzhou, Guangdong, China

**Keywords:** biomarker, immunotherapy, lung immune prognostic index, meta-analysis, urinary cancers

## Abstract

**Background:**

The Lung Immune Prognostic Index (LIPI), derived from the derived neutrophil-to-lymphocyte ratio and lactate dehydrogenase, integrates systemic inflammation and tumour burden. Its prognostic utility in urological malignancies has not been comprehensively quantified.

**Methods:**

We conducted a systematic review and meta-analysis in accordance with PRISMA and a prospectively registered protocol. PubMed, Embase, and the Cochrane Library were searched through October 2025. Eligible cohort studies or trials evaluated associations between LIPI categories (good/intermediate/poor) and survival outcomes in renal cell carcinoma, urothelial carcinoma, and prostate cancer. Random-effects models were fitted using REML with Hartung-Knapp adjustment. When hazard ratios (HRs) were not directly reported, we reconstructed pseudo-individual patient data from Kaplan-Meier curves to estimate HRs.

**Results:**

Thirteen studies (19 independent cohorts; 5,304 patients) were included. Compared with good LIPI, intermediate LIPI was associated with worse overall survival (OS) (HR 1.73, 95% CI 1.52-1.98) and shorter progression-free survival (PFS; including disease-free survival after surgery where applicable) (HR 1.39, 95% CI 1.13-1.71). Poor LIPI showed a stronger association with inferior OS (HR 3.73, 95% CI 2.95-4.71) and PFS (HR 2.69, 95% CI 1.96-3.70). Prognostic gradients were generally consistent across tumour types and treatment settings, including immune checkpoint inhibitor-treated cohorts.

**Conclusions:**

LIPI is a readily available biomarker that provides robust risk stratification across major urological cancers and may support treatment planning and trial stratification.

**Systematic Review Registration:**

https://www.crd.york.ac.uk/PROSPERO/view/CRD420251235082, identifier CRD420251235082.

## Introduction

Urological cancers, including prostate, bladder, kidney, urinary tract, testicular, and penile cancers, constitute a major component of the global cancer burden and are among the most common causes of cancer-related morbidity and mortality worldwide. Prostate cancer is currently the second most frequently diagnosed cancer among men globally, and its incidence is projected to double between 2020 and 2040 (1). Similarly, bladder cancer and renal cell carcinoma rank ninth and fourteenth respectively in global cancer incidence statistics (2–4). Although substantial progress has been made in the management of these tumors through approaches such as radical surgery, stereotactic body radiotherapy, immune checkpoint inhibitors (ICIs), and targeted molecular therapies, significant disparities in overall survival (OS) continue to exist across tumor subtypes, geographical regions, and treatment regimens (5, 6). As global cancer incidence rises with population aging and growth, there is an increasing demand for a reliable, low-cost, and clinically accessible biomarker to support pre-treatment risk stratification and facilitate personalized therapeutic decision-making.

Originally proposed in immunotherapy-treated non-small cell lung cancer (NSCLC) patients (7), the Lung Immune Prognostic Index (LIPI) correlates with disease control rates and ICI treatment outcomes, with significantly prolonged OS in the LIPI-good group compared to LIPI-poor group (median OS 15.6 months vs. 4.5 months) (8). Beyond the original development cohorts, a Frontiers-published systematic review and meta-analysis in ICI-treated NSCLC also confirmed a stepwise association between higher LIPI and worse OS/PFS, supporting the reproducibility of LIPI as a peripheral blood risk index in immunotherapy settings (9). Consequently, the distinct progression-free survival (PFS) and OS profiles across the good, intermediate, and poor LIPI groups enable preliminary prediction of immunotherapy efficacy. LIPI is developed based on a derived neutrophil-to-lymphocyte ratio (dNLR) greater than 3 and lactate dehydrogenase (LDH) levels exceeding the upper limit of normal (ULN). Elevated dNLR (≥3) indicates systemic inflammation and immune suppression, which are often associated with tumor immune escape (10–12). In parallel, elevated serum LDH levels reflect tumor hypoxia, Warburg effect, cellular necrosis, and glycolytic activity, signifying higher tumor burden and poor prognosis in cancer patients (13, 14).

Given the central role of systemic inflammation in the pathogenesis and progression of urological cancers, LIPI has recently been investigated as a potential prognostic biomarker beyond lung cancer. Retrospective studies in renal cell carcinoma (RCC) and advanced urothelial carcinoma (UC) have reported that patients with high pre-treatment LIPI scores experienced significantly worse OS and PFS following immunotherapy (15–17). Moreover, several studies suggest that the index may also identify patients at elevated risk of early relapse after surgery (18, 19). However, the current body of literature is constrained by limitations such as small sample sizes, heterogeneous endpoints, variable follow-up durations, and reliance on single-center datasets. These methodological inconsistencies contribute to mixed conclusions and limit the generalizability of findings. Furthermore, the comparative prognostic value of the LIPI relative to traditional models such as the International Metastatic Renal Cell Carcinoma Database Consortium (IMDC) risk score, the Bellmunt score, and the systemic immune-inflammation index remains uncertain.

To address these knowledge gaps, we conducted a comprehensive systematic review and meta-analysis to evaluate the prognostic performance of the LIPI in patients with urological cancers. This study aims to comprehensively evaluate the association between the LIPI and both OS and PFS across various tumor types and treatment modalities. It provides a high level of evidence supporting the clinical utility of the LIPI as a cost-effective and broadly applicable prognostic tool in the context of modern urological oncology.

## Methods

This systematic review and meta-analysis was conducted in accordance with the Preferred Reporting Items for Systematic Reviews and Meta-Analyses (PRISMA) guidelines (20). The protocol was prospectively registered on PROSPERO (No. CRD420251235082).

### Search strategy

Comprehensive literature searches were performed in PubMed, EMBASE, and the Cochrane Library databases to identify eligible studies evaluating the prognostic value of LIPI in urological malignancies published up to October 2025. The reference lists of all included articles were also manually screened to identify additional studies. The complete electronic search strategies for each database are provided in the **Supplementary Materials** (Search strategy).

### Outcomes

The primary objective of this meta-analysis was to evaluate the association between LIPI and survival outcomes in patients with urological tumors. The primary endpoint was OS, defined as the time from diagnosis or initiation of treatment to death from any cause. The secondary endpoints included PFS, disease-free survival (DFS), and cancer-specific survival (CSS). PFS was defined as the time from treatment initiation to documented disease progression or death, whichever occurred first, and was primarily applied to patients receiving systemic or pharmacologic therapy (e.g., immunotherapy, chemotherapy). DFS was defined as the time from curative-intent surgery to disease recurrence or death and was applicable to surgically treated cohorts. For the purpose of quantitative synthesis, DFS events in surgically treated patients were analyzed together with PFS events from medically treated cohorts, as both outcomes reflect time-to-progression endpoints and share comparable prognostic implications across different treatment modalities. Nevertheless, we recognize that pooling DFS and PFS may contribute to between-study heterogeneity. CSS was defined as the time from diagnosis or treatment to death specifically attributed to the urological malignancy, with deaths from other causes censored at the time of occurrence.

Based on the LIPI classification, patients were categorized as having a good LIPI score (score =0), intermediate (score = 1) or poor (score = 2). To comprehensively evaluate the prognostic impact of LIPI, we performed stratified comparisons across different LIPI groupings—specifically 0 vs. 1, 0 vs. 2, and 0 vs. (1 + 2)—to assess whether incremental deterioration in LIPI status was associated with worsening clinical outcomes.

### Inclusion and exclusion criteria

Eligibility criteria were defined using the Population, Intervention, Comparison, Outcome, and Study Design (PICOS) framework2. Studies were included if they met all of the following criteria: (1) Enrolled patients with histologically confirmed urological malignancies, including renal cell carcinoma, bladder cancer, prostate cancer, or upper tract urothelial carcinoma; (2) Investigated the prognostic significance of LIPI in relation to survival outcomes; (3) Reported at least one of the predefined endpoints (OS, PFS, DFS, or CSS); (4) Provided sufficient data to obtain or calculate hazard ratios (HRs) and 95% confidence intervals (CIs); (5) Were designed as clinical cohort studies or interventional trials with analyzable time-to-event data; and (6) Were full-text publications in English.

Exclusion criteria were as follows: (1) Reviews, editorials, commentaries, case reports, or conference abstracts lacking extractable data; (2) Studies that did not assess LIPI or lacked a clearly defined LIPI classification system; (3) Potential overlap was assessed by cross-checking data source (e.g., database/trial), participating institutions, recruitment periods, cancer type, and treatment setting. When overlap was suspected, we retained the report with the largest sample size and/or most comprehensive outcome reporting; (4) Preclinical studies involving animals or cell lines; and (5) Studies without sufficient data to estimate HRs and 95% CIs, even after contacting corresponding authors.

### Data extraction

Two reviewers independently extracted data from eligible studies, and discrepancies were resolved through discussion or consultation with a third investigator. Extracted data included: (1) general study characteristics, such as first author’s name, year of publication, country or region, study design (prospective or retrospective), cancer type, and total number of participants; (2) patient characteristics, including treatment modality (e.g., immune checkpoint inhibitors, chemotherapy, or targeted therapy), and follow-up duration; (3) definition and classification criteria of the LIPI; and (4) outcome measures, including OS, PFS, DFS, CSS, and the corresponding HRs with 95% CIs. When a single publication reported results for multiple clearly non-overlapping cohorts (e.g., distinct cancer types or treatment-defined populations), these cohorts were extracted and analyzed as independent units; when overlap was suspected, we retained the most informative dataset as prespecified.

When both univariate and multivariate HRs were available, results from multivariate analyses were preferentially extracted to minimize confounding. For studies not directly reporting HRs and 95% CIs, individual patient data (IPD) or pseudo-IPD were reconstructed from published Kaplan-Meier (KM) curves using Engauge Digitizer (version 11.1), following the methods described by Tierney et al. (2007) and Guyot et al. (2012) (21, 22). Briefly, survival probabilities were digitized from KM curves, and event counts were allocated to observed survival drops in proportion to their magnitudes. When numbers-at-risk or censoring indicators were reported, censoring was assigned to corresponding intervals; otherwise, censoring times were assumed to be uniformly distributed across consecutive time segments. The reconstructed time-to-event data were then analyzed using Cox proportional hazards models to estimate HRs and 95% CIs. This pseudo-IPD reconstruction enabled inclusion of studies that provided only graphical survival data. This process was performed independently by two reviewers, and discrepancies were resolved through discussion or adjudication by a third investigator. Reconstructed datasets and corresponding digitized curves are available from the corresponding author upon reasonable request.

### Quality assessment

The methodological quality of each included study was evaluated using the Newcastle-Ottawa Scale (NOS), which assesses three domains: selection of participants, comparability of study groups, and outcome assessment. Studies with NOS scores ≥7 were classified as high quality, 5–6 as moderate quality, and <5 as low quality.

### Statistical analyses

All statistical analyses were performed using R software (version 4.3.0; R Foundation for Statistical Computing, Vienna, Austria) with the meta and metafor packages. HRs with corresponding 95% CIs were used as summary effect sizes. Between-study heterogeneity was quantified using Cochran’s Q test and the I² statistic, representing the proportion of total variation due to heterogeneity rather than chance. Substantial heterogeneity was defined as I² > 50% or p < 0.10 for the Q test. The meta-analysis was performed using a random-effects model with the restricted maximum likelihood (REML) estimator for between-study variance (τ²), and the Hartung-Knapp adjustment was applied to provide more robust and conservative confidence intervals. For comparison, results from the fixed-effects model were also reported to assess the robustness of the pooled estimates. Random-effects models were prespecified as the primary approach to account for anticipated clinical and methodological heterogeneity, while fixed-effect models were evaluated only as sensitivity analyses. Forest plots were generated to display pooled HRs and 95% CIs. Subgroup analyses were conducted according to the type of HR (univariate vs multivariate), cancer type, treatment modality, and study quality (NOS score) to explore potential sources of heterogeneity. Sensitivity analyses were performed by sequentially omitting one study at a time (“leave-one-out” approach) to assess the influence of individual studies on overall estimates. Potential publication bias was evaluated using funnel plots, Egger’s regression test, and Begg’s rank correlation test. When asymmetry suggested possible bias, the trim-and-fill method was applied to estimate the potential effect of unpublished studies. All statistical tests were two-sided, and p < 0.05 was considered statistically significant.

## Results

### Study selection

**Figure 1:**
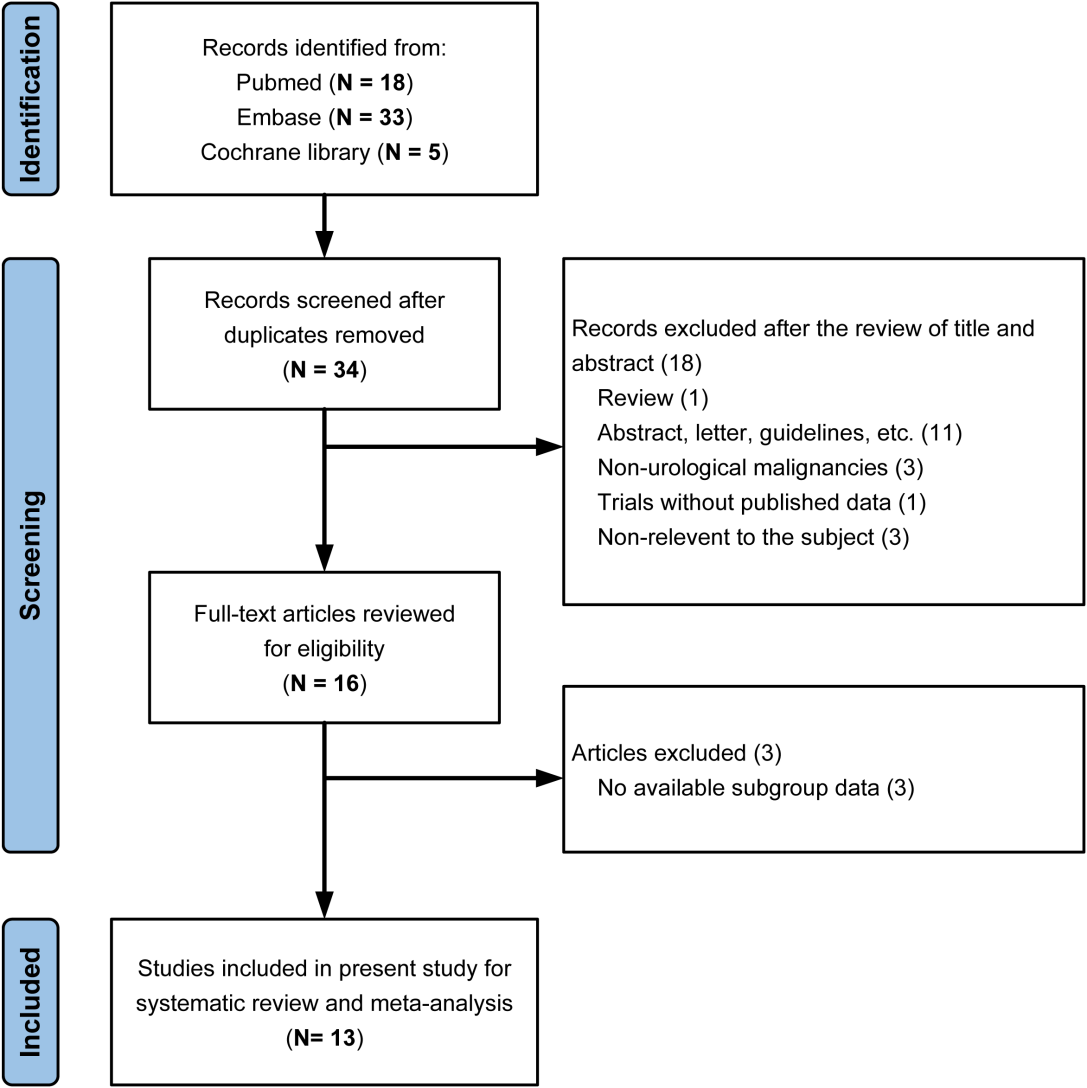
Study selection process based on the PRISMA guidelines. The flowchart depicts the systematic selection process for studies included in this meta-analysis. Records were identified through database searching and screened for eligibility according to predefined inclusion and exclusion criteria. After removing duplicates and screening titles/abstracts, 13 studies (representing 19 cohorts) were included in the final quantitative synthesis.

A total of 56 records were identified, and 34 unique articles were screened after removing duplicates. 13 eligible studies comprising 19 independent, non-overlapping cohorts met the inclusion criteria and were included in the quantitative synthesis (**Figure 1**) (15–19, 23–30).

### Study characteristics

13 studies represented 19 independent cohorts and collectively enrolled 5,304 patients, comprising five RCC (15, 23–25, 30), six UC (16–19, 26, 27), and two prostate cancer (PCA) studies (28, 29). Overall, 3051 patients had good LIPI and 1902 had intermediate or poor LIPI. Most cohorts involved patients receiving ICIs or targeted therapies, while five evaluated surgically treated populations (**Table 1**). Study quality was generally high (median NOS score: 7; **Supplementary Table 1**).

**Table 1:** Baseline characteristics of included studies.

Study ID	Author	Publication year	Study design	Region	Study period	Cancer type	Number of patients				Treatment regimen	Outcomes	NOS
							Overall	LIPI = 0	LIPI = 1	LIPI = 2			
1	Hoshina	2025	Single-center, R	Japan	2014-2024	RCC	80	60	16	4	Surgical (partial or total resection)	OS	5
2	Shibata	2024	Single-center, R	Japan	2012-2021	UC	567	263	253	51	Surgical (radical nephroureterectomy)	OS,DFS,CSS	7
3-1	Kobayashi	2024	Multi-center, R	Japan	2017-2022	UC	243	125	91	18	ICI (avelumab or pembrolizumab)	OS,PFS	7
3-2	Kobayashi	2024	Multi-center, R	Japan	2017-2022	UC	35	24	11	0	ICI (avelumab)	OS	7
3-3	Kobayashi	2024	Multi-center, R	Japan	2017-2022	UC	199	101	80	18	ICI (pembrolizumab)	OS	7
4	Ou	2024	Single-center, R	China	2017-2021	UC	81	43	27	11	Surgical (radical nephroureterectomy and cystsleeve resection)	OS	5
5-1	Wang	2024	Single-center, R	China	2005-2022	PCA	407	206	142	59	Maximal androgen blockade	OS,PFS	8
5-2	Wang	2024	Single-center, R	China	2005-2022	PCA	158	70	69	19	Abiraterone plus castration therapy	OS,PFS	8
6	Yamashita	2024	Multi-center, R	Japan	2015-2022	RCC	156	84	52	20	ICI (nivolumab plus ipilimumab)	OS,PFS,CSS	8
7-1	Carril-Ajuria	2024	Multi-center, P	Europe	2016-2017	RCC	619	364	216	39	ICI (nivolumab)	OS,PFS	8
7-2	Carril-Ajuria	2024	Multi-center, P	Europe	2008-2009	RCC	159	119	40*		VEGF/VEGFR-targeted therapy	OS,PFS	8
7-3	Carril-Ajuria	2024	Multi-center, P	Mainly Europe	2014-2016	RCC	542	404	138*		ICI (nivolumab plus ipilimumab)	OS,PFS	8
7-4	Carril-Ajuria	2024	Multi-center, P	Mainly Europe	2014-2016	RCC	542	411	131*		Sunitinib	OS,PFS	8
8	Ishiyama	2024	Multi-center, R	Japan	2004-2021	RCC	235	119	91	25	Surgical (radical nephrectomy)	DFS	9
9	Nakamura	2023	Multi-center, R	Japan	2018-2022	UC	90	41	33	16	ICI (pembrolizumab after platinum-based chemotherapy)	OS,PFS	7
10-1	Parent	2023	Multi-center, R	France	2015-2019	UC	137	77	48	12	ICI (not combinations of immunotherapy plus chemotherapy)	OS,PFS	8
10-2	Parent	2023	Multi-center, P	Mainly Europe	2016-2018	UC	541	281	198	63	ICI (atezolizumab)	OS,PFS	8
10-3	Parent	2023	Multi-center, R	France	2011-2019	UC	67	39	21	7	Chemotherapy	OS,PFS	8
11	Obayashi	2022	Single-center, R	Japan	2013-2019	UC	105	71	34*		Surgical (Radical cystectomy with extended pelvic lymph node dissection)	OS,DFS,CSS	6
12	Yamada	2020	Single-center, R	Japan	2007-2018	PCA	196	59	95	42	ADT based treatment	OS,CSS	7
13	Meyers	2019	Multi-center, R	Canada	2010-2019	RCC	145	90	46	9	ICI (nivolumab, pembrolizumab, ipilimumab plus nivolumab)	OS,PFS	9

*Patients with a LIPI score of 1 and 2 were combined into a single group.

LIPI, lung immune prognostic index; NOS, Newcastle-Ottawa Scale; RCC, renal cell carcinoma; UC, urothelial carcinoma; PCA, prostate cancer; R, retrospective study; P, prospective study; ICI, immune checkpoint inhibitor; VEGF/VEGFR, vascular endothelial growth factor/VEGF receptor; ADT, androgen deprivation therapy; OS, overall survival; DFS, disease-free survival; CSS, cancer-specific survival; PFS, progression-free survival.

### Pooled analysis of OS

#### Good vs. intermediate LIPI

**Figure 2:**
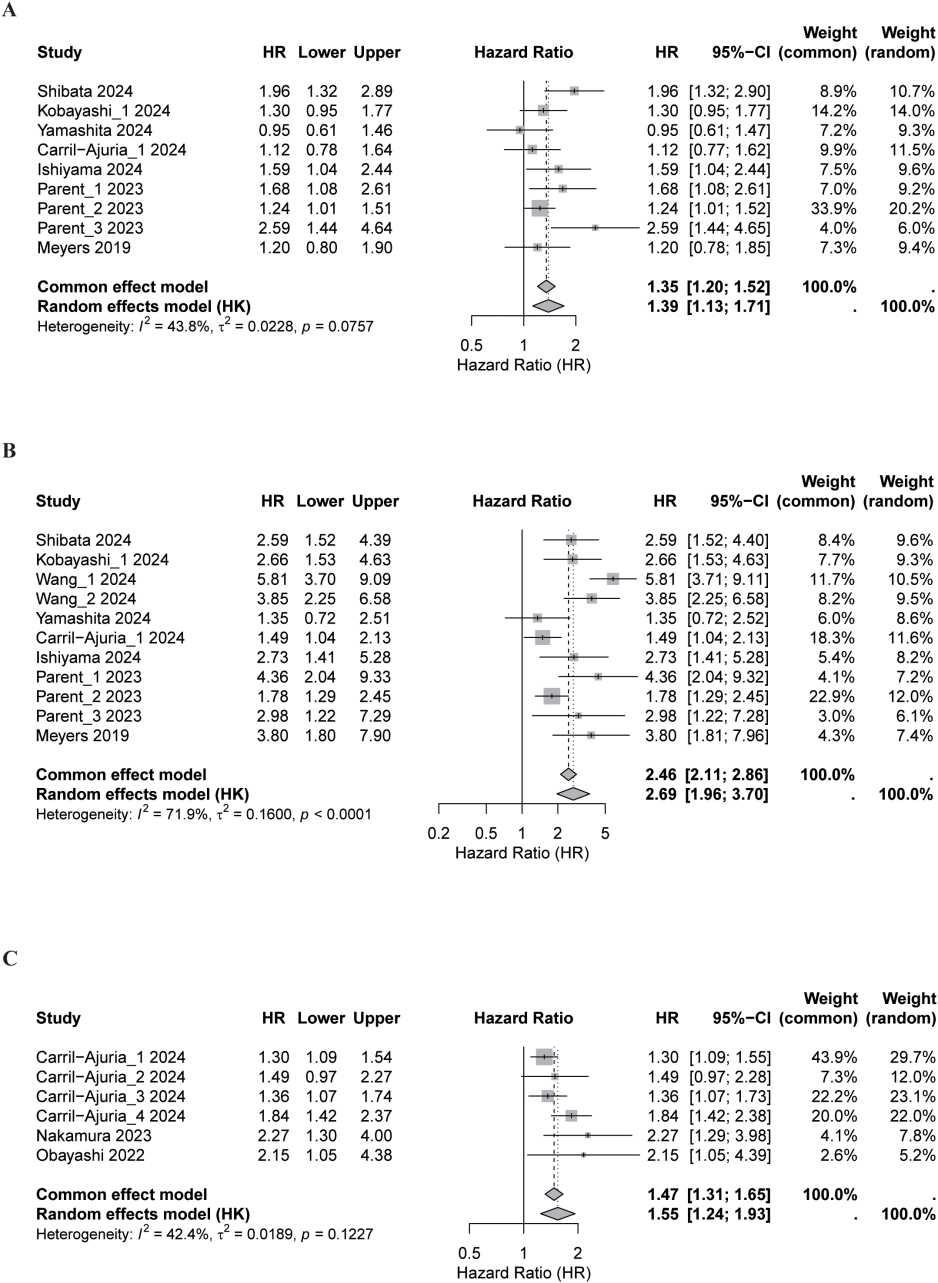
Forest plots of pooled overall survival (OS) according to Lung Immune Prognostic Index (LIPI) status. Meta-analytic comparisons were performed for **(A)** good vs. intermediate LIPI, **(B)** good vs. poor LIPI, and **(C)** good vs. intermediate or poor LIPI. Both Random-effects and common effect models were applied to estimate pooled hazard ratios (HRs) and 95% confidence intervals (CIs). The size of each square corresponds to the weight of the study, and the diamond represents the overall pooled estimate.

Across 11 cohorts, intermediate LIPI was significantly associated with worse OS compared with good LIPI (HR, 1.73; 95% CI, 1.52-1.98), with no observed heterogeneity (*I*² = 0.0%; **Figure 2**). Subgroup analyses demonstrated consistent effects across cancer types, HR sources, and study quality (**Supplementary Figure 1**), with stronger associations among ICIs-treated patients (p = 0.026) and surgically treated cohorts (p < 0.001).

**Figure 3:**
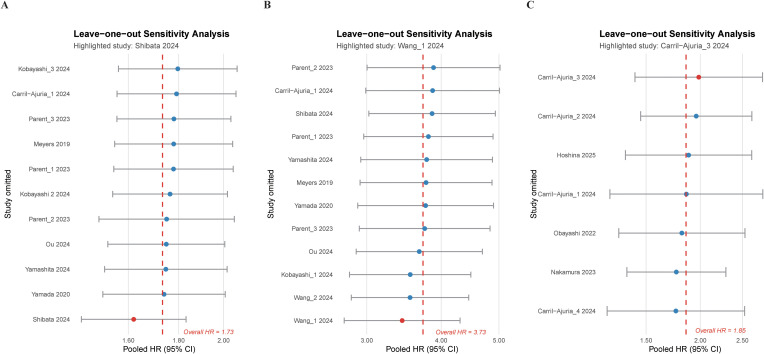
Leave-one-out sensitivity analysis assessing the robustness of pooled overall survival (OS) estimates across different Lung Immune Prognostic Index (LIPI) comparisons. Panels **(A-C)** illustrate the results of sequentially omitting one study at a time to evaluate the influence of individual studies on the pooled hazard ratio (HR) for OS in comparisons of **(A)** good vs. intermediate LIPI, **(B)** good vs. poor LIPI, and **(C)** good vs. intermediate or poor LIPI. The dashed horizontal line represents the overall pooled HR, while dots indicate recalculated HRs after each study’s exclusion. The minimal variation across panels demonstrates the stability and reliability of the pooled estimates.

Sensitivity analyses confirmed the stability of the pooled estimate (**Figure 3**). No significant publication bias was observed (Begg’s p = 0.533; Egger’s p = 0.136), and trim-and-fill analysis yielded an adjusted HR consistent with the primary result (**Supplementary Figure 2**). Meta-regression identified no significant moderators apart from treatment modality (**Supplementary Table 2**).

#### Good vs. poor LIPI

Poor LIPI was strongly associated with inferior OS compared with good LIPI across 12 cohorts (HR, 3.73; 95% CI, 2.95-4.71), with modest heterogeneity (*I*² = 31.6%; **Figure 2**). Subgroup analyses demonstrated broadly consistent associations across study-level factors, while suggesting some effect-size variation by cancer type, with larger estimates in PCA and UC and comparatively smaller estimates in RCC (p < 0.001; **Supplementary Figure 3**).

Sensitivity analyses supported result robustness (**Figure 3**). Publication bias assessments indicated no significant asymmetry (Begg’s p = 0.451; Egger’s p = 0.735), and trim-and-fill adjustment produced similar estimates (**Supplementary Figure 4**). Meta-regression supported cancer type as a significant effect modifier; relative to the reference category, the association was attenuated in RCC and UC (β = −0.69 and −0.61, respectively; both p < 0.05; **Supplementary Table 2**).

#### Good vs. intermediate or poor LIPI

Seven cohorts compared good LIPI with intermediate or poor categories. Unfavorable LIPI was associated with significantly higher mortality (HR, 1.85; 95% CI, 1.38-2.49), with moderate heterogeneity (*I*² = 50.8%, p = 0.058; **Figure 2**). Subgroup analyses showed consistent directional effects across study characteristics (**Supplementary Figure 5**), though variability, particularly within UC, indicated reduced precision (p < 0.001).

Sensitivity analyses confirmed stability (**Figure 3**), and neither publication bias (Egger’s p = 0.864) nor trim-and-fill procedures meaningfully altered findings (**Supplementary Figure 6**). Meta-regression did not identify significant moderators (**Supplementary Table 2**).

### Pooled analysis of PFS

#### Good vs. intermediate LIPI

**Figure 4:**
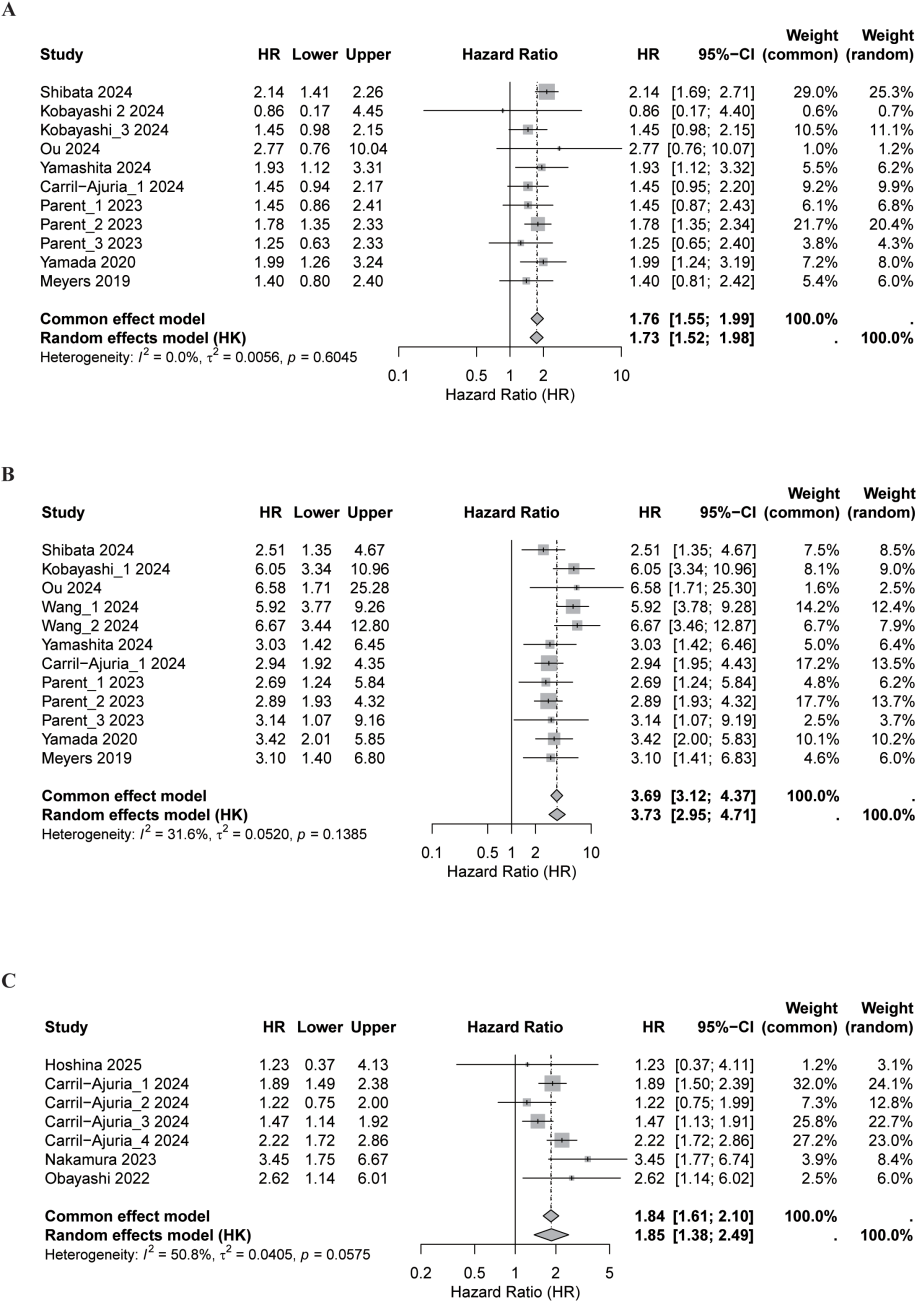
Forest plots of pooled progression-free survival (PFS) according to Lung Immune Prognostic Index (LIPI) status. Meta-analytic comparisons were performed for **(A)** good vs. intermediate LIPI, **(B)** good vs. poor LIPI, and **(C)** good vs. intermediate or poor LIPI. Both Random-effects and common effect models were applied to estimate pooled hazard ratios (HRs) and 95% confidence intervals (CIs). The size of each square corresponds to the weight of the study, and the diamond represents the overall pooled estimate.

Nine cohorts reported PFS outcomes. Intermediate LIPI was associated with shorter PFS (HR, 1.39; 95% CI, 1.13-1.71), with moderate heterogeneity (*I*² = 43.8%; **Figure 4**). Subgroup analyses showed consistent patterns across most study characteristics, with weaker associations among ICIs-treated patients (p = 0.001) and stronger effects in surgically managed cohorts (p = 0.013; **Supplementary Figure 7**).

Sensitivity analyses supported estimate stability (**Supplementary Figure 8A**). No publication bias was detected (Begg’s p = 0.348; Egger’s p = 0.225), and trim-and-fill analysis identified no missing studies (**Supplementary Figure 9**). Meta-regression identified ICIs treatment as the only significant modifier (**Supplementary Table 3**).

#### Good vs. poor LIPI

Across 11 cohorts, poor LIPI was associated with markedly shorter PFS (HR, 2.69; 95% CI, 1.96-3.70), with substantial heterogeneity (*I*² = 71.9%, p < 0.001; **Figure 4**). Subgroup analyses revealed variability by cancer type and treatment regimen, with stronger associations in UC and PCA (p = 0.007) and in non-ICIs cohorts (p = 0.038; **Supplementary Figure 10**).

Sensitivity analyses demonstrated robustness (**Supplementary Figure 8B**). Meta-regression identified cancer type and ICI status as contributors to heterogeneity (**Supplementary Table 3**). Publication bias assessments indicated no meaningful asymmetry (Begg’s p = 0.213; Egger’s p = 0.167), and trim-and-fill analyses yielded consistent estimates (**Supplementary Figure 11**).

#### Good vs. intermediate or poor LIPI

Six cohorts compared PFS across combined intermediate/poor categories versus good LIPI. Higher LIPI scores were associated with shorter PFS (HR, 1.55; 95% CI, 1.24-1.93), with moderate but non-significant heterogeneity (*I*² = 42.4%; **Figure 4**). Subgroup findings were generally consistent, though effect sizes were stronger among non-ICIs-treated patients and in UC (**Supplementary Figure 12**). Meta-regression suggested that cancer type and study quality contributed to variability but were not statistically significant (**Supplementary Table 3**). Sensitivity analyses confirmed the stability of results (**Supplementary Figure 8C**). No publication bias (Begg’s p = 0.260; Egger’s p = 0.106) was detected, and trim-and-fill estimates supported robustness (**Supplementary Figure 13**).

### Pooled analysis of cancer-specific survival

#### Good vs. intermediate LIPI

Three cohorts reported cancer-specific survival (CSS) outcomes for patients with good versus intermediate LIPI. Intermediate LIPI was associated with a higher risk of cancer-specific mortality, although the confidence interval crossed unity (HR, 2.05; 95% CI, 0.92-4.57), and the heterogeneity was low (*I*² = 17.5%; **Supplementary Figure 14A**). Sensitivity analyses showed that removal of any single study did not materially change the pooled estimate (**Supplementary Figure 15A**). No significant publication bias was detected (Egger’s p = 0.288). Trim-and-fill analysis imputed two hypothetical studies, producing an adjusted HR of 2.80 (95% CI, 1.48-5.30), which remained directionally consistent with the primary analysis.

#### Good vs. poor LIPI

Three cohorts compared CSS outcomes for patients with good versus poor LIPI. Poor LIPI was strongly associated with higher cancer-specific mortality (HR, 3.58; 95% CI, 3.18-4.05; **Supplementary Figure 14B**). Sensitivity analyses demonstrated that sequential exclusion of individual cohorts did not materially affect the pooled estimate (**Supplementary Figure 15B**). Egger’s test detected no evidence of publication bias (p = 0.823), and trim-and-fill analysis imputed one hypothetical study, yielding an adjusted HR (3.51; 95% CI, 3.19-3.87) that remained consistent with the primary result.

However, given the small number of cohorts, formal tests for publication bias are underpowered in this setting and should be interpreted cautiously.

## Discussion

Recent studies have explored the prognostic role of LIPI in renal cell carcinoma, urothelial carcinoma, and prostate cancer, confirming that poor LIPI is closely associated with significantly reduced OS and PFS. This meta-analysis, incorporating 13 studies and 5,304 patients, indicates that poor LIPI predicts worse clinical outcomes, including OS, PFS, and CSS, with consistent directionality of this prognostic association across RCC, UC, and PCa. Pooled OS analysis revealed significantly increased mortality risk in patients with intermediate or poor LIPI compared to those with good LIPI (LIPI-int HR = 1.73; LIPI-poor HR = 3.73). Notably, the prognostic effect was most pronounced in PCa (LIPI-poor vs LIPI-good HR = 6.15), followed by UC (HR = 3.42) and RCC (HR = 3.10), suggesting LIPI may reflect underlying tumor-specific immune interactions or biological aggressiveness. A similar pattern emerged for PFS. The larger effect size observed in PCa may, in part, reflect disease-specific resistance biology in advanced stages, where sustained intratumoral androgens, AR pathway reactivation, and genetic AR alterations contribute to aggressive phenotypes under treatment pressure (31). Consistent with this concept, AR mutations such as T877A and W741L can alter ligand binding and are being explored as targets for anti-androgen strategies (32). Among ICIs-treated patients, those with intermediate or poor LIPI exhibited significantly increased disease progression risk compared to those with good LIPI (PFS LIPI-int HR = 1.23; LIPI-poor HR = 2.15; LIPI int/poor HR = 1.36). However, these associations were more pronounced in the cohort not receiving ICIs (PFS LIPI-int HR = 1.92; LIPI-poor HR = 3.95; LIPI int/poor HR = 1.77), potentially reflecting an amplified impact of systemic inflammation and tumor burden on prognosis in the absence of immune modulation. Similarly, patients undergoing surgical treatment exhibited a more pronounced prognostic gradient, suggesting baseline LIPI may reflect not only tumor biology but also perioperative immune function and systemic resistance. Although fewer studies have explored CSS, trends were consistent with the above indicators. Patients with intermediate LIPI showed a slightly increased CSS risk (HR = 2.05), while those with poor LIPI exhibited a significantly elevated risk (HR = 3.58). These findings were consistent across different cohorts with low heterogeneity and remained robust after sensitivity analyses and publication bias tests, further validating LIPI’s value as a reliable long-term tumor prognosis biomarker. This meta-analysis highlights LIPI’s clinical significance in risk stratification and individualized treatment planning for patients with urological malignancies.

Chronic inflammation plays a central role in tumorigenesis and cancer progression, influencing tumor cell proliferation, angiogenesis, immune evasion, and metastasis (33, 34). Elevated dNLR reflects a systemic inflammatory state characterized by neutrophilia and lymphopenia, which is associated with immunosuppressive tumor microenvironments, reduced antitumor immune surveillance, and poor clinical outcomes (35–38). Neutrophils promote tumor development through several mechanisms, including the release of proangiogenic factors, recruitment of immunosuppressive cells, secretion of extracellular matrix-degrading enzymes, formation of NETs and induction of EMT (39–41). Conversely, lymphopenia limits cytotoxic T cell activity, disrupts immune homeostasis, and contributes to resistance to therapies such as ICIs (42–44). LDH, as the key enzyme catalyzing the interconversion of lactate and pyruvate, serves as a crucial indicator reflecting tumor metabolic activity and tumor burden (45). Elevated LDH levels reflect increased glycolytic activity, hypoxia-induced necrosis, and immunosuppression within the tumor microenvironment (46). These elevations are strongly associated with more aggressive disease, shorter survival, and increased risk of metastasis across various solid tumors (e.g., melanoma (47), lung cancer (48), colorectal cancer (49), prostate cancer (50), etc.) and hematologic malignancies (51). Mechanistically, glycolytic rewiring can couple lactate biology to epigenetic programs implicated in therapy tolerance; in bladder cancer, a glycolysis-histone lactylation (H3K9la) axis has been reported in the context of cisplatin resistance (52). The value of LIPI lies in its dual representation of host immune status (dNLR) and tumor aggressiveness (LDH). This dual-axis framework provides a more holistic assessment of host-tumor interactions than either biomarker alone.

In lung cancers, LIPI has been widely validated as an independent predictor of survival across treatment modalities, including chemotherapy, targeted therapy, and immunotherapy (53–56). In NSCLC patients receiving ICIs, poor LIPI correlates with significantly shorter survival outcomes (57). Similar findings in small cell lung cancer support its broader histological relevance (53). The present analysis confirms that these associations extend to urological malignancies, reinforcing the potential of LIPI as a cross-tumor, immunologically anchored prognostic tool. Although previous single-institution or cohort studies provided valuable insights, they were limited by specific cohort biases and lacked statistical power to support broader conclusions across different treatment regimens and cancer subtypes. For example, Carril-Ajuria et al. (23) demonstrated in a prospective cohort that among mRCC patients treated with ICIs, LIPI was significantly associated with OS (LIPI-good 30.1 vs 13.8 months in the LIPI int/poor; HR = 0.47) and PFS (HR = 0.74), but did not perform cross-validation across different treatment types or tumor subtypes. Similarly, Parent et al. (16) reported that poor LIPI in mUC patients predicted worse survival, though the wide confidence interval indicated limited statistical certainty. In contrast, this meta-analysis improves statistical reliability through pooled hazard ratios, subgroup analyses, and sensitivity testing. These approaches confirm the prognostic consistency of LIPI across different urological tumor types and treatment strategies. Compared to existing models such as the Bellmunt score, LIPI offers a practical advantage (16). It is based on two routine laboratory parameters, complete blood count and serum biochemistry, and does not require imaging or subjective clinical assessment. Although the IMDC risk score especially valuable for stratifying risk in mRCC, LIPI provides complementary and independent prognostic information (23). Nevertheless, LIPI has some limitations including susceptibility to non-tumor-related inflammation and lack of refinement regarding tumor microenvironment biology and clinical variables such as performance status and anemia. For example, microbiota perturbations and antibiotic exposure can reshape antitumor immunity and immunotherapy efficacy in urological tumors, potentially influencing inflammatory indices and contributing to residual between-cohort variability (58). In addition, circadian regulation of immune responses and the tumor microenvironment may introduce temporal variability in host inflammatory states, highlighting the importance of standardized timing of laboratory measurements in future prospective validation (59).

This meta-analysis emphasizes the practical relevance of LIPI as a cost-effective, objective, and rapid tool for risk stratification in patients with urological cancers. It may assist clinicians in identifying high-risk patients who require more intensive monitoring or tailored therapeutic strategies. Beyond clinical practice, LIPI offers value as a stratification tool in prospective clinical trials, enabling more balanced randomization and enrichment strategies for immunotherapy responders. This may be particularly relevant as urological oncology moves toward precision, biomarker-informed trial designs and combination regimens in UC, where pragmatic peripheral indices could complement molecular profiling for enrollment stratification (60). Because LIPI relies on readily available laboratory values, it is especially useful in resource-constrained settings where access to molecular profiling or advanced imaging is limited. Therefore, LIPI represents a scalable and accessible approach to personalized cancer care across diverse healthcare environments. Beyond peripheral inflammatory indices, emerging tumor-associated immune targets may provide complementary stratification layers; for example, B7-H3 (CD276) shows broad and temporally stable expression in metastatic tumor specimens and is being clinically explored as an immunotherapeutic target, supporting future composite models that integrate systemic inflammation with tumor-intrinsic immune features (61). In parallel, non-apoptotic cell-death programs (e.g., paraptosis) have been proposed as complementary strategies to bypass resistance in urological malignancies, providing additional context for why inflammation- and metabolism-linked biomarkers may track aggressive, therapy-refractory states (62). Looking ahead, peripheral indices such as LIPI may be complemented by emerging immunotherapy-relevant biomarker layers derived from the “dark proteome,” including noncanonical proteins encoded by previously overlooked genomic regions. Advances in ribosome profiling, mass spectrometry, and proteogenomics have highlighted noncanonical proteins as potential biomarker and neoantigen sources, supporting future composite stratification strategies that integrate systemic inflammation with tumor-intrinsic immune targets (63). Future prospective studies may also evaluate LIPI alongside liquid-biopsy readouts, such as circulating tumor cells (including clusters), which provide a minimally invasive window into metastatic dissemination, cellular plasticity, and treatment-relevant tumor evolution (64).

This is the first systematic review and meta-analysis to comprehensively evaluate the prognostic value of the LIPI across all major urological malignancies, including renal cell carcinoma, urothelial carcinoma, and prostate cancer. The study adheres strictly to the PRISMA guidelines, ensuring transparency, methodological rigor, and reproducibility. By aggregating data from over 5,000 patients across 13 studies and 19 cohorts, this analysis provides robust pooled estimates with high statistical power. The inclusion of detailed subgroup analyses by tumor type, treatment modality (e.g., immune checkpoint inhibitors, surgery), and risk stratification level enhances the reliability and granularity of the findings. Nonetheless, several limitations warrant consideration. Most included studies were retrospective in nature, introducing potential biases such as confounding and selection bias. Although heterogeneity was generally low in key pooled analyses, variation in patient characteristics, staging, treatment protocols, and follow-up durations may limit the generalizability of the findings. Notably, heterogeneity was most pronounced for PFS when comparing poor versus good LIPI, likely reflecting multiple sources of between-study diversity. The included cohorts encompassed distinct urological malignancies (RCC, UC, and PCA), which differ in baseline prognosis and underlying biology. Treatment context also varied substantially, spanning ICI-treated populations, non-ICI systemic regimens, and surgically managed cohorts; consistent with this, subgroup analyses and meta-regression suggested that cancer type and ICI status contributed to between-study variability. In addition, time-to-event endpoints were not fully harmonized across studies. To maximize available evidence, DFS from surgical settings was pooled with PFS from medically treated cohorts as a time-to-progression construct; however, differences in clinical context and endpoint definitions may have inflated heterogeneity and should be considered when interpreting PFS-related pooled estimates. Importantly, despite these sources of variability, the direction of the association remained broadly consistent across most strata, supporting the overall robustness of the findings. Additionally, the absence of individual patient data (IPD) precluded more nuanced time-to-event analyses and adjustment for confounders such as PD-L1 expression or performance status. Pseudo-IPD reconstructed from KM curves may introduce measurement uncertainty due to digitization error and censoring assumptions, although this approach enables inclusion of otherwise eligible evidence. Finally, while publication bias was formally tested and found to be minimal, the possibility of unreported negative studies cannot be entirely excluded.

In conclusion, this systematic review and meta-analysis confirms that LIPI is a clinically significant and independent prognostic biomarker in patients with urological cancers. By capturing both systemic inflammation and tumor burden, LIPI provides a reliable and accessible method to predict survival outcomes across diverse therapeutic contexts. Future large-scale prospective studies are warranted to further validate its prognostic value, assess its predictive relevance in patients receiving immunotherapy or targeted treatments, and explore its integration with molecular, clinical, and pathological markers to inform personalized management strategies in urological oncology.

## Data Availability

The original contributions presented in the study are included in the article/**Supplementary Material**. Further inquiries can be directed to the corresponding authors.
