# Supplementary material for: The prognostic value of the lung immune prognostic index in patients with urological cancers: a systematic review and meta-analysis
Source: Front Immunol. 2026 Mar 25;17:1806105. doi: 10.3389/fimmu.2026.1806105 (PMC13057338; doi:10.3389/fimmu.2026.1806105)
Supplement: Supplementary file 1 [file Table1.docx]

**Supplementary Materials**

**Search Strategy (Until October, 2025)**

**Graphical Abstract.** Association of the lung immune prognostic index with survival outcomes in urological cancers

**Supplementary Figure 1.** Subgroup analyses of overall survival (OS) comparing patients with good versus intermediate Lung Immune Prognostic Index (LIPI).

**Supplementary Figure 2.** Publication bias evaluation for overall survival (OS) comparing good versus intermediate Lung Immune Prognostic Index (LIPI).

**Supplementary Figure 3.** Subgroup analyses of overall survival (OS) comparing patients with good versus poor Lung Immune Prognostic Index (LIPI).

**Supplementary Figure 4.** Publication bias evaluation for overall survival (OS) comparing good versus poor Lung Immune Prognostic Index (LIPI).

**Supplementary Figure 5.** Subgroup analyses of overall survival (OS) comparing patients with good versus intermediate or poor Lung Immune Prognostic Index (LIPI).

**Supplementary Figure 6.** Publication bias evaluation for overall survival (OS) comparing good versus intermediate or poor Lung Immune Prognostic Index (LIPI).

**Supplementary Figure 7.** Subgroup analyses of progress-free survival (PFS) comparing patients with good versus intermediate Lung Immune Prognostic Index (LIPI).

**Supplementary Figure 8.** Leave-one-out sensitivity analysis assessing the robustness of pooled progress-free survival (PFS) estimates across different Lung Immune Prognostic Index (LIPI) comparisons.

**Supplementary Figure 9.** Publication bias evaluation for progress-free survival (PFS) comparing good versus intermediate Lung Immune Prognostic Index (LIPI).

**Supplementary Figure 10.** Subgroup analyses of progress-free survival (PFS) comparing patients with good versus poor Lung Immune Prognostic Index (LIPI).

**Supplementary Figure 11.** Publication bias evaluation for progress-free survival (PFS) comparing good versus poor Lung Immune Prognostic Index (LIPI).

**Supplementary Figure 12.** Subgroup analyses of progress-free survival (PFS) comparing patients with good versus intermediate or poor Lung Immune Prognostic Index (LIPI).

**Supplementary Figure 13.** Publication bias evaluation for progress-free survival (PFS) comparing good versus intermediate or poor Lung Immune Prognostic Index (LIPI).

**Supplementary Figure 14.** Forest plots of pooled cancer specific survival (CSS) according to Lung Immune Prognostic Index (LIPI) status.

**Supplementary Figure 15.** Leave-one-out sensitivity analysis assessing the robustness of pooled cancer specific survival (CSS) estimates across different Lung Immune Prognostic Index (LIPI) comparisons.

**Supplementary Table 1.** Methodological quality assessment of included studies using the Newcastle–Ottawa Scale.

**Supplementary Table 2.** Meta-regression analysis of potential moderators affecting the pooled hazard ratios (HRs) for overall survival.

**Supplementary Table 3.** Meta-regression analysis of potential moderators affecting the pooled hazard ratios (HRs) for progression-free survival.

**Search Strategy (Until October, 2025):**

**Pubmed (2025.10.19):**

1."lung immune prognostic index"[Title/Abstract] (130)

2."LIPI"[Title/Abstract] (308)

3.#1 OR #2 (328)

4."Urinary Bladder Neoplasms"[Mesh] (67804)

5.bladder cancer (106643)

6.urothelial carcinoma (36539)

7."Carcinoma, Transitional Cell"[Mesh] (22860)

8."Kidney Neoplasms"[Mesh] (90420)

9.renal cell carcinoma (72332)

10.RCC (27619)

11.kidney cancer (163744)

12."Prostatic Neoplasms"[Mesh] (161151)

13.prostate cancer (232137)

14."Ureteral Neoplasms"[Mesh] (5390)

15.ureteral cancer (11037)

16.#4 OR #5 OR #6 OR #7 OR #8 OR #9 OR #10 OR #11 OR #12 OR #13 OR #14 OR #15 (502470)

17.#3 AND #16 (18)

**Embase by OVID (2025.10.16):**

1. 'lung immune prognostic index'.ab,ti. (247)
2. LIPI.ab,ti. (484)
3. 1 OR 2 (507)
4. exp urinary bladder cancer/ (102996)
5. 'bladder cancer'.af. (102384)
6. 'urothelial carcinoma'.af. (25717)
7. exp transitional cell carcinoma/ (45760)
8. exp kidney neoplasms/ (194868)
9. 'renal cell carcinoma'.af. (94264)
10. RCC.af. (40447)
11. 'kidney cancer'.af. (37304)
12. exp prostate cancer/ (309208)
13. 'prostatic neoplasm'.af. (728)
14. exp ureteral cancer/ (2597)
15. 'ureteral carcinoma'.af. (258)
16. 4 OR 5 OR 6 OR 7 OR 8 OR 9 OR 10 OR 11 OR 12 OR 13 OR 14 OR 15 (615253)

17.3 AND 16 (33)

**Cochrane Library (2025.10.19):**

1. "lung immune prognostic index":ti,ab (15)
2. "LIPI":ti,ab (170)
3. 1 OR 2 (174)
4. [mh "Urinary Bladder Neoplasms"] (2467)
5. bladder cancer (6890)
6. urothelial carcinoma (1502)
7. [mh "Carcinoma, Transitional Cell"] (899)
8. [mh "Kidney Neoplasms"] (2049)
9. renal cell carcinoma (4876)
10. RCC (2468)
11. kidney cancer (8214)
12. [mh "Prostatic Neoplasms"] (9182)
13. prostate cancer (20561)
14. [mh "Ureteral Neoplasms"] (44)
15. ureteral cancer (203)
16. 4 OR 5 OR 6 OR 7 OR 8 OR 9 OR 10 OR 11 OR 12 OR 13 OR 14 OR 15(36856
17. 3 AND 16 (5)

**
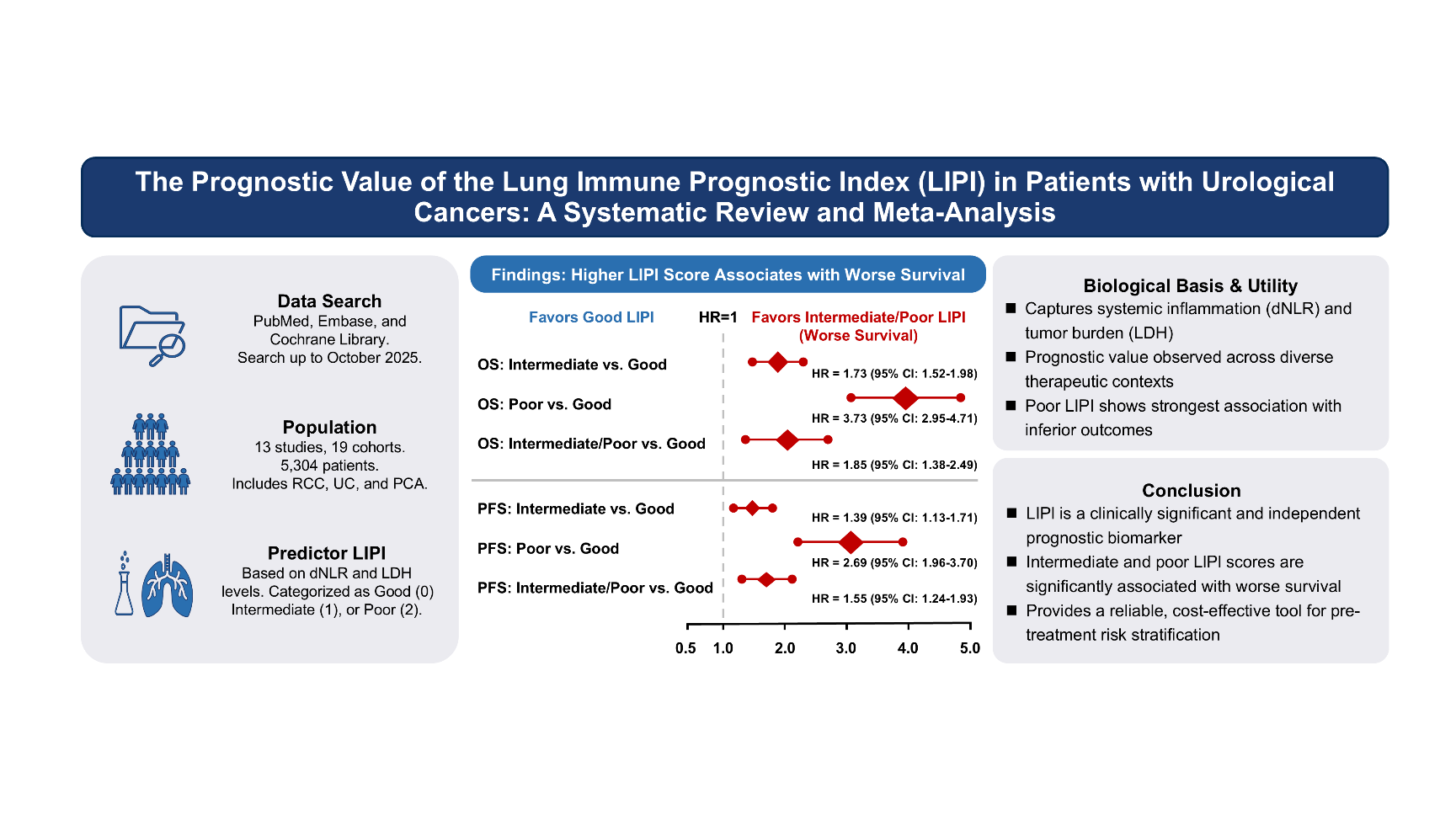
Graphical Abstract. Association of the lung immune prognostic index with survival outcomes in urological cancers.**

This systematic review and meta-analysis of 13 studies involving 5,304 patients evaluates the prognostic value of the Lung Immune Prognostic Index (LIPI) in urological malignancies. The graphical abstract summarizes the data sources, study population, and forest plots demonstrating that intermediate and poor pre-treatment LIPI scores are significantly associated with worse survival outcomes compared with good LIPI scores.

LIPI, Lung Immune Prognostic Index; RCC, renal cell carcinoma; UC, urothelial carcinoma; PCA, prostate cancer; dNLR, derived neutrophil-to-lymphocyte ratio; LDH, lactate dehydrogenase; HR, hazard ratio; OS, overall survival; PFS, progression-free survival.

**Supplementary Figure 1. Subgroup analyses of overall survival (OS) comparing patients with good versus intermediate Lung Immune Prognostic Index (LIPI).**

Panels (A–E) summarize subgroup meta-analyses assessing whether the prognostic association between LIPI and OS varied according to (A) cancer type, (B) hazard ratios (HR) type (univariate vs. multivariate), (C) study quality as assessed by the Newcastle–Ottawa Scale (NOS), (D) immune checkpoint inhibitor (ICI) treatment status, and (E) surgical treatment. Horizontal lines indicate 95% confidence intervals (CIs) for each subgroup, and diamonds represent pooled HRs. The largely consistent direction and magnitude of HRs across subgroups suggest that the prognostic impact of LIPI on OS is robust across different study and patient characteristics.

**Supplementary Figure 2. Publication bias evaluation for overall survival (OS) comparing good versus intermediate Lung Immune Prognostic Index (LIPI).**

(A) Funnel plot of the included studies demonstrates visual assessment of potential publication bias. (B) Funnel plot adjusted by the trim-and-fill method, showing imputed studies (open circles) to estimate the potential effect of missing data. (C) Forest plot displaying the adjusted pooled hazard ratio (HR) and 95% confidence interval (CI) after applying the trim-and-fill method.

**Supplementary Figure 3. Subgroup analyses of overall survival (OS) comparing patients with good versus poor Lung Immune Prognostic Index (LIPI).**

Panels (A–E) summarize subgroup meta-analyses assessing whether the prognostic association between LIPI and OS varied according to (A) cancer type, (B) hazard ratios (HR) type (univariate vs. multivariate), (C) study quality as assessed by the Newcastle–Ottawa Scale (NOS), (D) immune checkpoint inhibitor (ICI) treatment status, and (E) surgical treatment. Horizontal lines indicate 95% confidence intervals (CIs) for each subgroup, and diamonds represent pooled HRs.

**Supplementary Figure 4. Publication bias evaluation for overall survival (OS) comparing good versus poor Lung Immune Prognostic Index (LIPI).**

(A) Funnel plot of the included studies demonstrates visual assessment of potential publication bias. (B) Funnel plot adjusted by the trim-and-fill method, showing imputed studies (open circles) to estimate the potential effect of missing data. (C) Forest plot displaying the adjusted pooled hazard ratio (HR) and 95% confidence interval (CI) after applying the trim-and-fill method.

**Supplementary Figure 5. Subgroup analyses of overall survival (OS) comparing patients with good versus intermediate or poor Lung Immune Prognostic Index (LIPI).**

Panels (A–E) summarize subgroup meta-analyses assessing whether the prognostic association between LIPI and OS varied according to (A) cancer type, (B) hazard ratios (HR) type (univariate vs. multivariate), (C) study quality as assessed by the Newcastle–Ottawa Scale (NOS), (D) immune checkpoint inhibitor (ICI) treatment status, and (E) surgical treatment. Horizontal lines indicate 95% confidence intervals (CIs) for each subgroup, and diamonds represent pooled HRs.

**Supplementary Figure 6. Publication bias evaluation for overall survival (OS) comparing good versus intermediate or poor Lung Immune Prognostic Index (LIPI).**

(A) Funnel plot of the included studies demonstrates visual assessment of potential publication bias. (B) Funnel plot adjusted by the trim-and-fill method, showing imputed studies (open circles) to estimate the potential effect of missing data. (C) Forest plot displaying the adjusted pooled hazard ratio (HR) and 95% confidence interval (CI) after applying the trim-and-fill method.

**Supplementary Figure 7. Subgroup analyses of progress-free survival (PFS) comparing patients with good versus intermediate Lung Immune Prognostic Index (LIPI).**

Panels (A–D) summarize subgroup meta-analyses assessing whether the prognostic association between LIPI and PFS varied according to (A) cancer type, (B) hazard ratios (HR) type (univariate vs. multivariate), (C) immune checkpoint inhibitor (ICI) treatment status, and (D) surgical treatment. Horizontal lines indicate 95% confidence intervals (CIs) for each subgroup, and diamonds represent pooled HRs.

**Supplementary Figure 8. Leave-one-out sensitivity analysis assessing the robustness of pooled progress-free survival (PFS) estimates across different Lung Immune Prognostic Index (LIPI) comparisons.**

Panels (A–C) illustrate the results of sequentially omitting one study at a time to evaluate the influence of individual studies on the pooled hazard ratio (HR) for PFS in comparisons of (A) good vs. intermediate LIPI, (B) good vs. poor LIPI, and (C) good vs. intermediate or poor LIPI. The dashed horizontal line represents the overall pooled HR, while dots indicate recalculated HRs after each study’s exclusion. The minimal variation across panels demonstrates the stability and reliability of the pooled estimates.

**Supplementary Figure 9. Publication bias evaluation for progress-free survival (PFS) comparing good versus intermediate Lung Immune Prognostic Index (LIPI).**

(A) Funnel plot of the included studies demonstrates visual assessment of potential publication bias. (B) Funnel plot adjusted by the trim-and-fill method, showing imputed studies (open circles) to estimate the potential effect of missing data. (C) Forest plot displaying the adjusted pooled hazard ratio (HR) and 95% confidence interval (CI) after applying the trim-and-fill method.

**Supplementary Figure 10. Subgroup analyses of progress-free survival (PFS) comparing patients with good versus poor Lung Immune Prognostic Index (LIPI).**

Panels (A–D) summarize subgroup meta-analyses assessing whether the prognostic association between LIPI and PFS varied according to (A) cancer type, (B) hazard ratios (HR) type (univariate vs. multivariate), (C) immune checkpoint inhibitor (ICI) treatment status, and (D) surgical treatment. Horizontal lines indicate 95% confidence intervals (CIs) for each subgroup, and diamonds represent pooled HRs.

**Supplementary Figure 11. Publication bias evaluation for progress-free survival (PFS) comparing good versus poor Lung Immune Prognostic Index (LIPI).**

(A) Funnel plot of the included studies demonstrates visual assessment of potential publication bias. (B) Funnel plot adjusted by the trim-and-fill method, showing imputed studies (open circles) to estimate the potential effect of missing data. (C) Forest plot displaying the adjusted pooled hazard ratio (HR) and 95% confidence interval (CI) after applying the trim-and-fill method.

**Supplementary Figure 12. Subgroup analyses of progress-free survival (PFS) comparing patients with good versus intermediate or poor Lung Immune Prognostic Index (LIPI).**

Panels (A–D) summarize subgroup meta-analyses assessing whether the prognostic association between LIPI and PFS varied according to (A) cancer type, (B) hazard ratios (HR) type (univariate vs. multivariate), (C) immune checkpoint inhibitor (ICI) treatment status, and (D) surgical treatment. Horizontal lines indicate 95% confidence intervals (CIs) for each subgroup, and diamonds represent pooled HRs.

**Supplementary Figure 13. Publication bias evaluation for progress-free survival (PFS) comparing good versus intermediate or poor Lung Immune Prognostic Index (LIPI).**

(A) Funnel plot of the included studies demonstrates visual assessment of potential publication bias. (B) Funnel plot adjusted by the trim-and-fill method, showing imputed studies (open circles) to estimate the potential effect of missing data. (C) Forest plot displaying the adjusted pooled hazard ratio (HR) and 95% confidence interval (CI) after applying the trim-and-fill method.

**Supplementary Figure 14. Forest plots of pooled cancer specific survival (CSS) according to Lung Immune Prognostic Index (LIPI) status.**

Meta-analytic comparisons were performed for (A) good vs. intermediate LIPI and (B) good vs. poor LIPI. Both Random-effects and common effect models were applied to estimate pooled hazard ratios (HRs) and 95% confidence intervals (CIs). The size of each square corresponds to the weight of the study, and the diamond represents the overall pooled estimate.

**Supplementary Figure 15. Leave-one-out sensitivity analysis assessing the robustness of pooled cancer specific survival (CSS) estimates across different Lung Immune Prognostic Index (LIPI) comparisons.**

Panels illustrate the results of sequentially omitting one study at a time to evaluate the influence of individual studies on the pooled hazard ratio (HR) for CSS in comparisons of (A) good vs. intermediate LIPI and (B) good vs. poor LIPI. The dashed horizontal line represents the overall pooled HR, while dots indicate recalculated HRs after each study’s exclusion. The minimal variation across panels demonstrates the stability and reliability of the pooled estimates.

**Supplementary Table 1. Methodological quality assessment of included studies using the Newcastle–Ottawa Scale.**

**Supplementary Table 2. Meta-regression analysis of potential moderators affecting the pooled hazard ratios (HRs) for overall survival.**

**Supplementary Table 3. Meta-regression analysis of potential moderators affecting the pooled hazard ratios (HRs) for progression-free survival.**


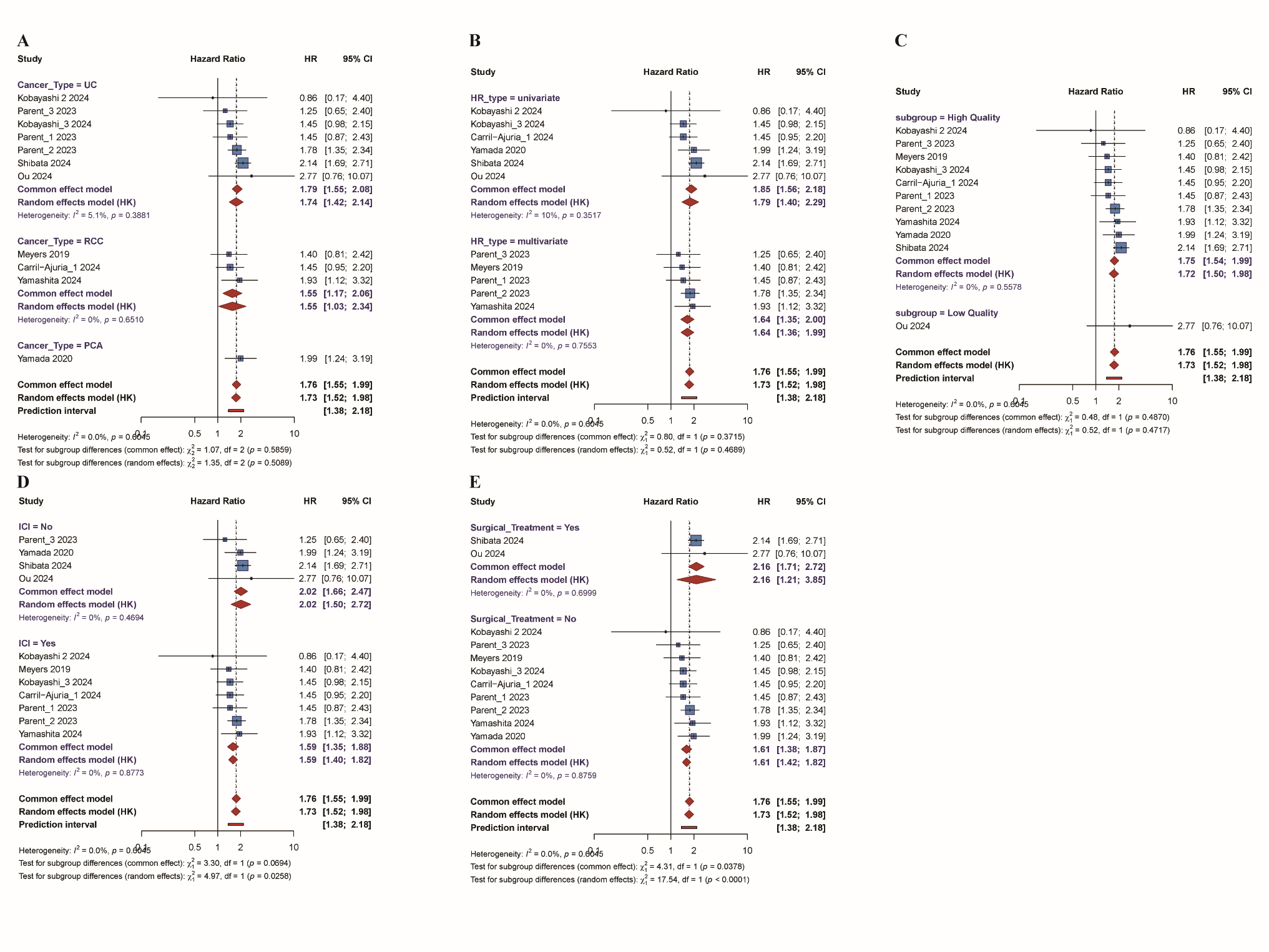


**Supplementary Figure 1. Subgroup analyses of overall survival (OS) comparing patients with good versus intermediate Lung Immune Prognostic Index (LIPI).**

Panels (A–E) summarize subgroup meta-analyses assessing whether the prognostic association between LIPI and OS varied according to (A) cancer type, (B) hazard ratios (HR) type (univariate vs. multivariate), (C) study quality as assessed by the Newcastle–Ottawa Scale (NOS), (D) immune checkpoint inhibitor (ICI) treatment status, and (E) surgical treatment. Horizontal lines indicate 95% confidence intervals (CIs) for each subgroup, and diamonds represent pooled HRs. The largely consistent direction and magnitude of HRs across subgroups suggest that the prognostic impact of LIPI on OS is robust across different study and patient characteristics.

**
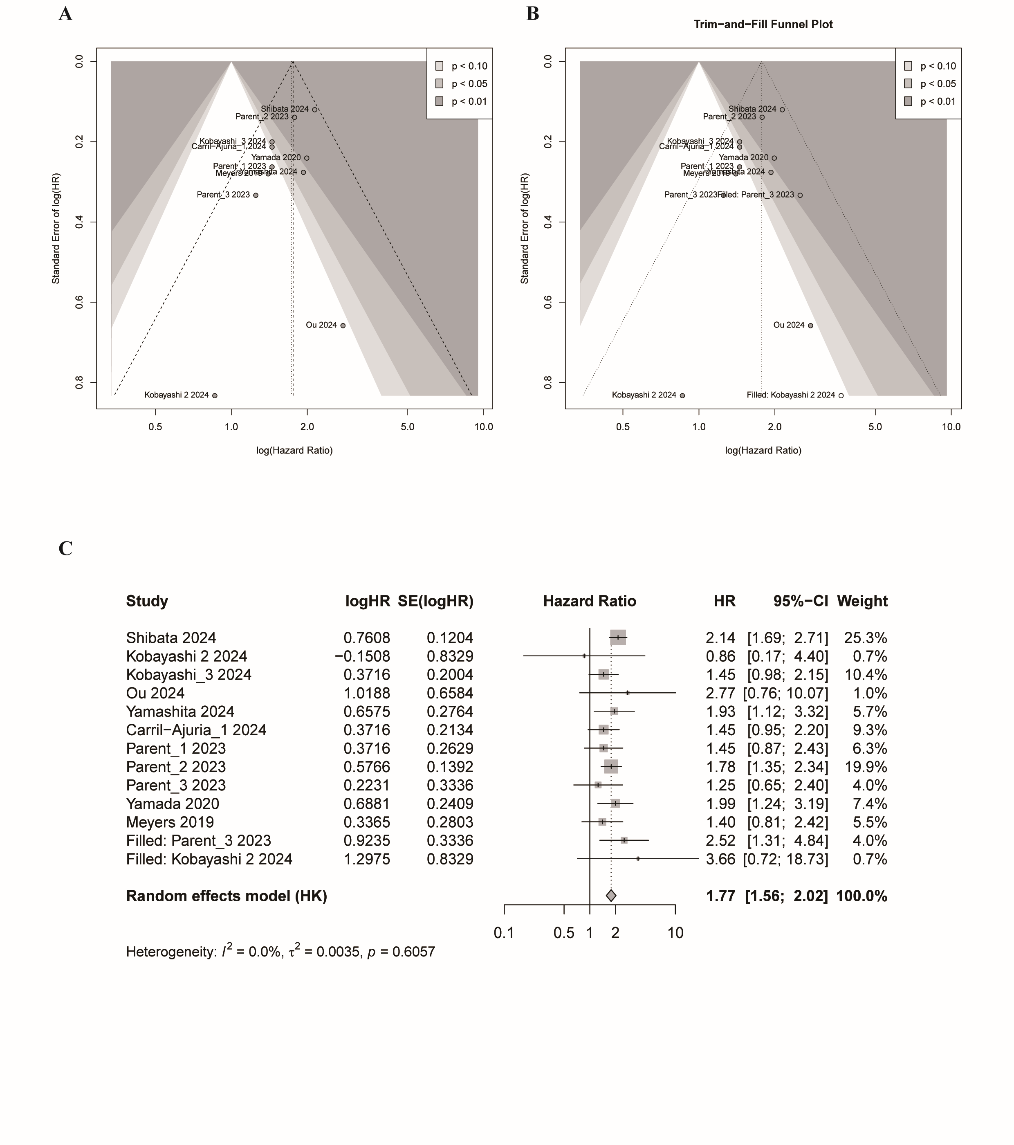
**

**Supplementary Figure 2. Publication bias evaluation for overall survival (OS) comparing good versus intermediate Lung Immune Prognostic Index (LIPI).**

(A) Funnel plot of the included studies demonstrates visual assessment of potential publication bias. (B) Funnel plot adjusted by the trim-and-fill method, showing imputed studies (open circles) to estimate the potential effect of missing data. (C) Forest plot displaying the adjusted pooled hazard ratio (HR) and 95% confidence interval (CI) after applying the trim-and-fill method.


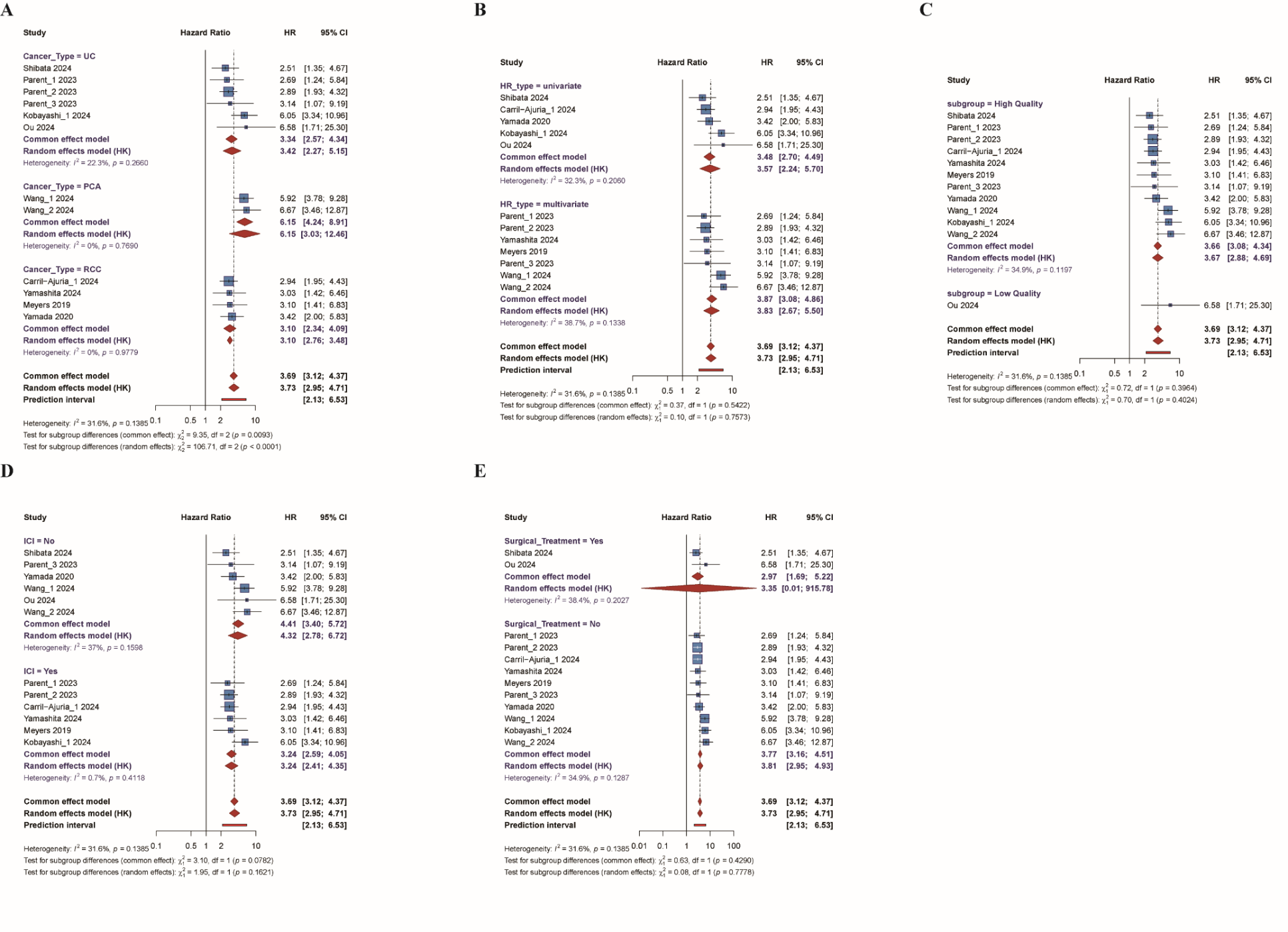


**Supplementary Figure 3. Subgroup analyses of overall survival (OS) comparing patients with good versus poor Lung Immune Prognostic Index (LIPI).**

Panels (A–E) summarize subgroup meta-analyses assessing whether the prognostic association between LIPI and OS varied according to (A) cancer type, (B) hazard ratios (HR) type (univariate vs. multivariate), (C) study quality as assessed by the Newcastle–Ottawa Scale (NOS), (D) immune checkpoint inhibitor (ICI) treatment status, and (E) surgical treatment. Horizontal lines indicate 95% confidence intervals (CIs) for each subgroup, and diamonds represent pooled HRs.


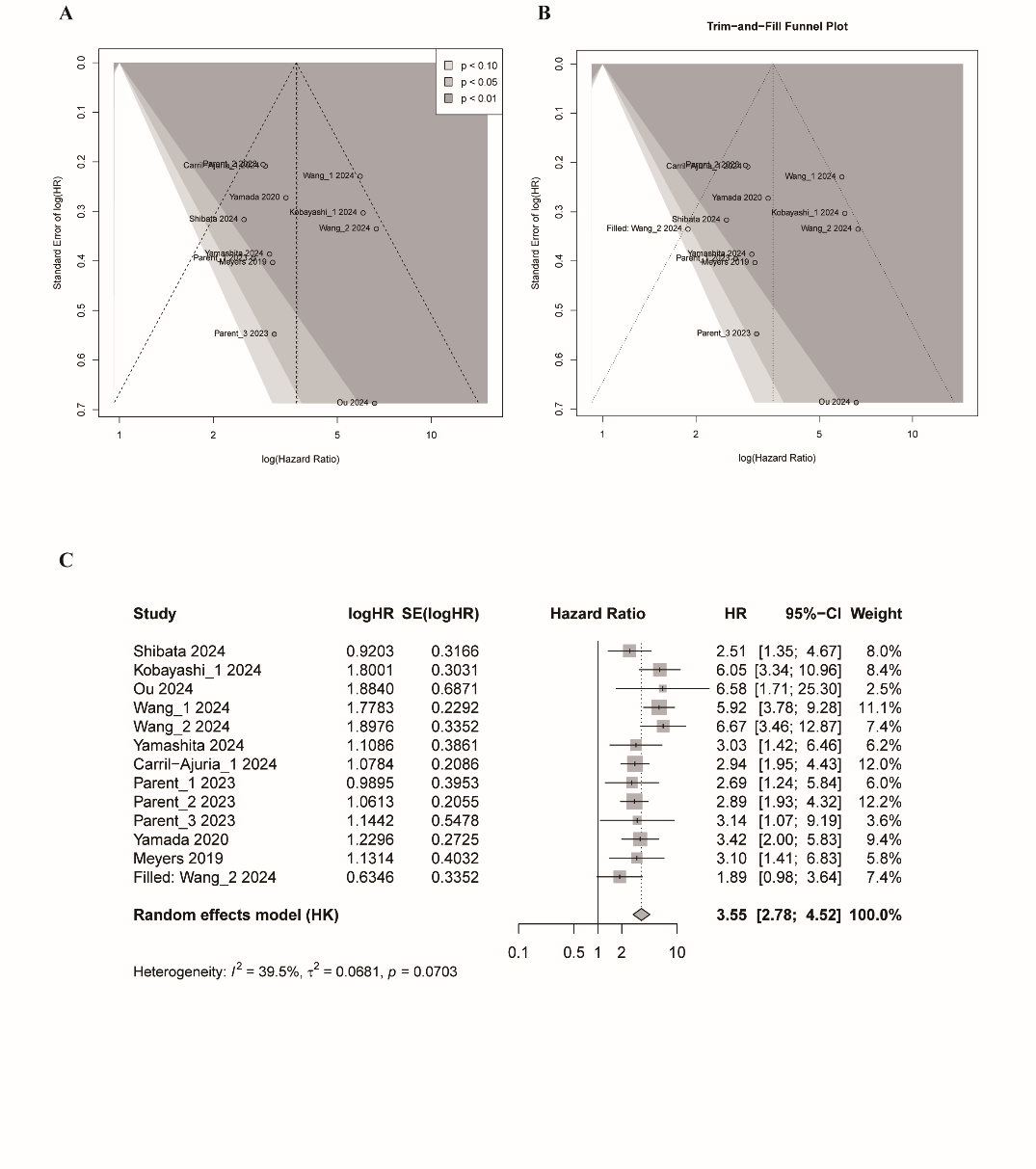


**Supplementary Figure 4. Publication bias evaluation for overall survival (OS) comparing good versus poor Lung Immune Prognostic Index (LIPI).**

(A) Funnel plot of the included studies demonstrates visual assessment of potential publication bias. (B) Funnel plot adjusted by the trim-and-fill method, showing imputed studies (open circles) to estimate the potential effect of missing data. (C) Forest plot displaying the adjusted pooled hazard ratio (HR) and 95% confidence interval (CI) after applying the trim-and-fill method.

**
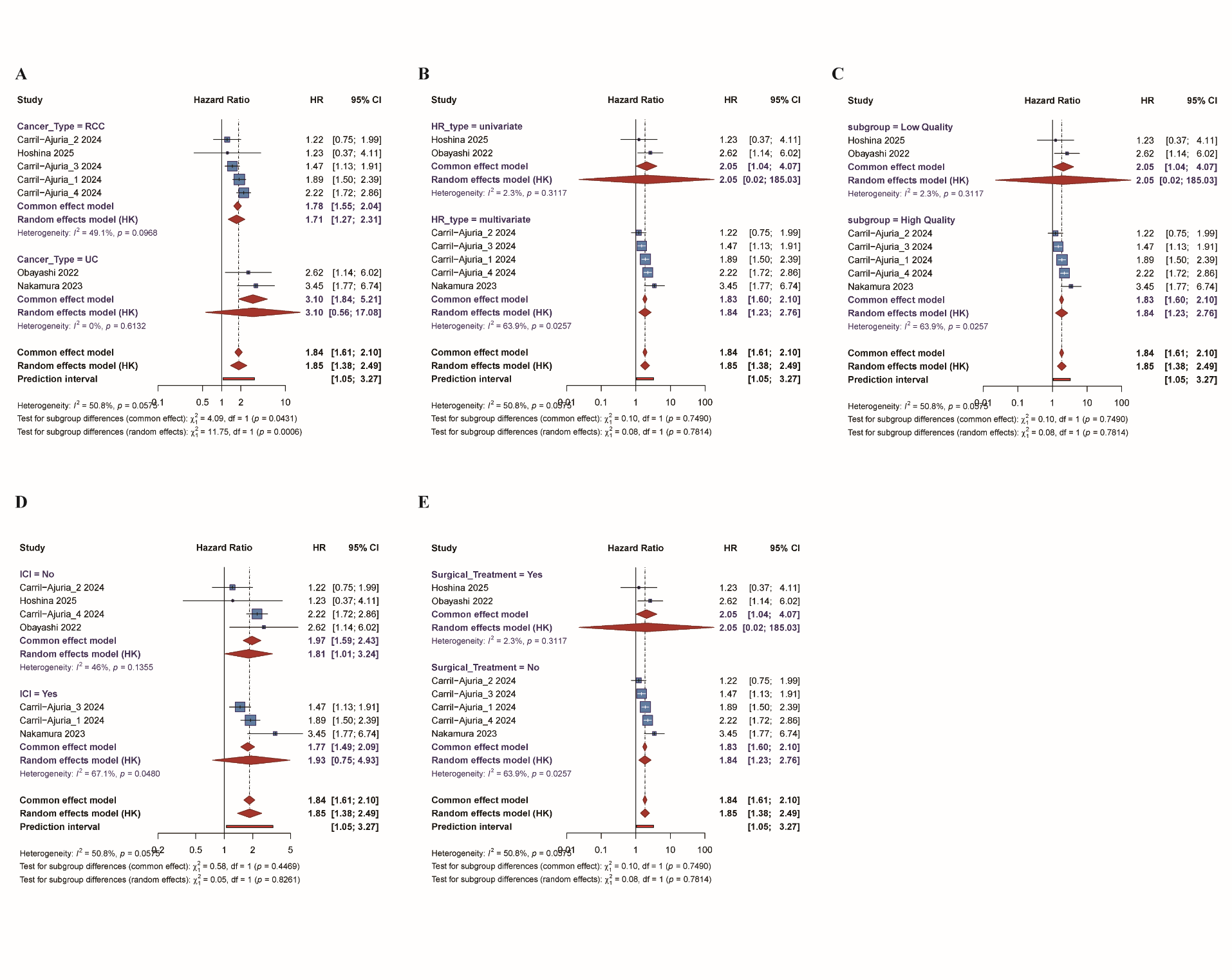
**

**Supplementary Figure 5. Subgroup analyses of overall survival (OS) comparing patients with good versus intermediate or poor Lung Immune Prognostic Index (LIPI).**

Panels (A–E) summarize subgroup meta-analyses assessing whether the prognostic association between LIPI and OS varied according to (A) cancer type, (B) hazard ratios (HR) type (univariate vs. multivariate), (C) study quality as assessed by the Newcastle–Ottawa Scale (NOS), (D) immune checkpoint inhibitor (ICI) treatment status, and (E) surgical treatment. Horizontal lines indicate 95% confidence intervals (CIs) for each subgroup, and diamonds represent pooled HRs.

**
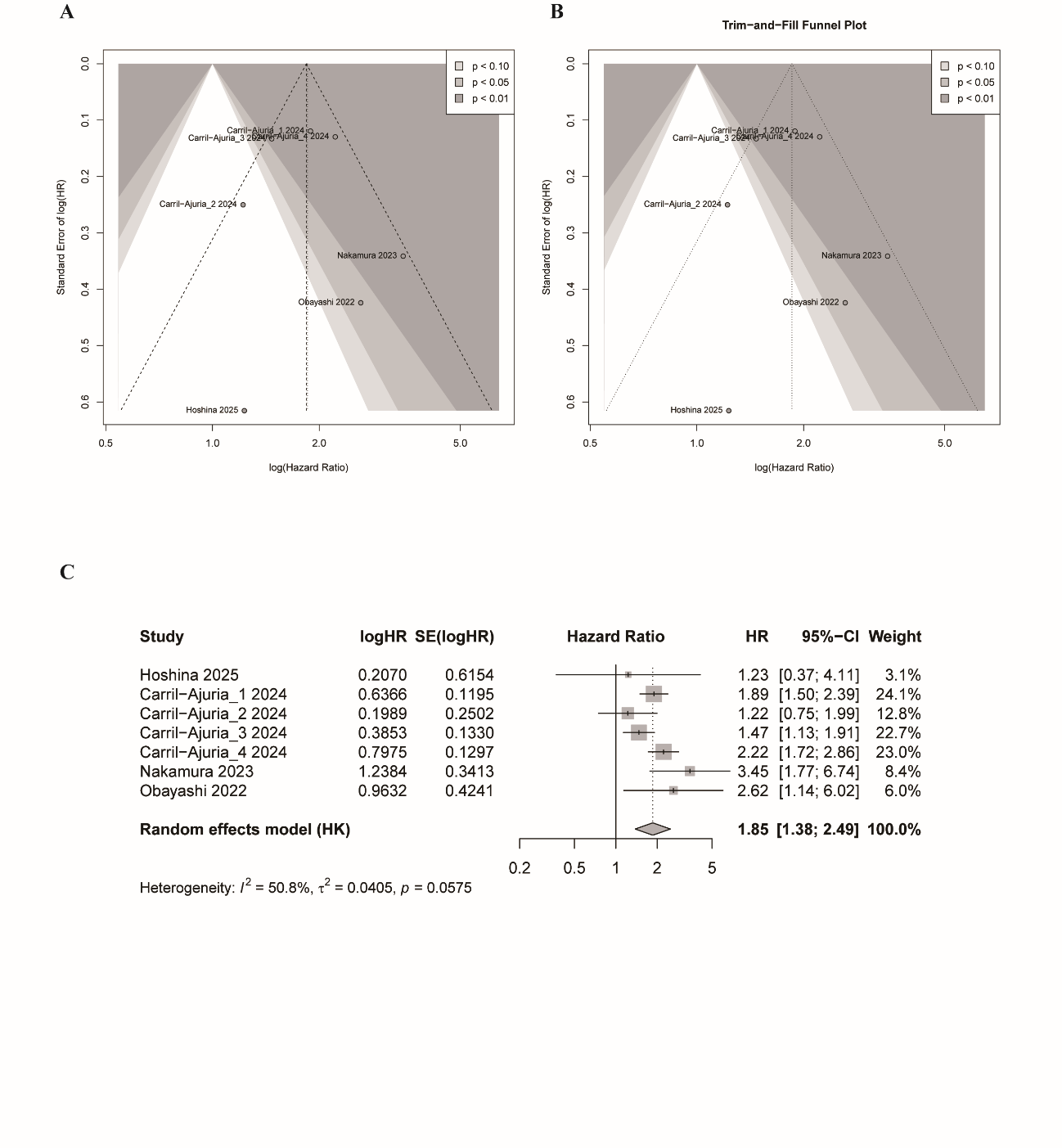
**

**Supplementary Figure 6. Publication bias evaluation for overall survival (OS) comparing good versus intermediate or poor Lung Immune Prognostic Index (LIPI).**

(A) Funnel plot of the included studies demonstrates visual assessment of potential publication bias. (B) Funnel plot adjusted by the trim-and-fill method, showing imputed studies (open circles) to estimate the potential effect of missing data. (C) Forest plot displaying the adjusted pooled hazard ratio (HR) and 95% confidence interval (CI) after applying the trim-and-fill method.


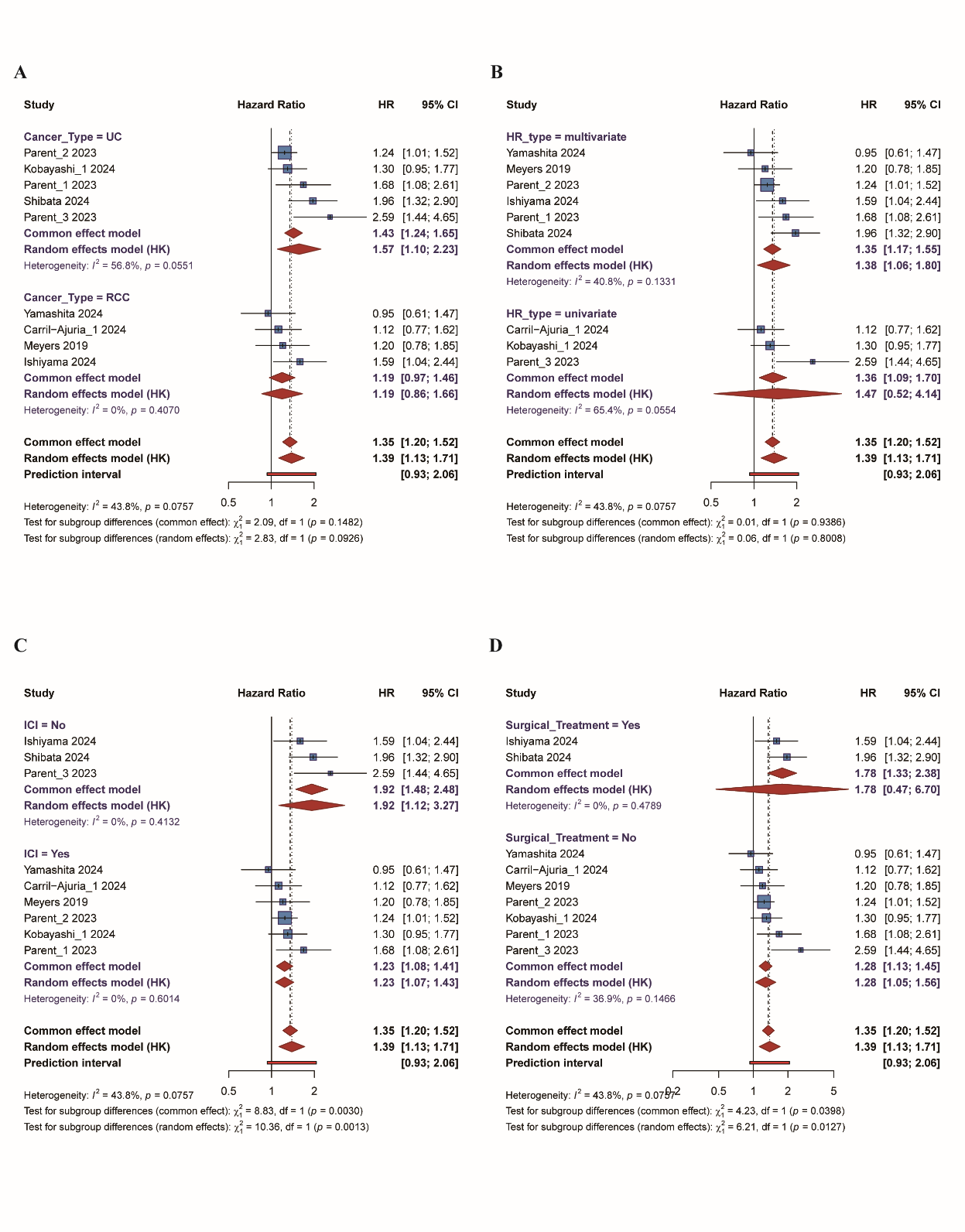


**Supplementary Figure 7. Subgroup analyses of progress-free survival (PFS) comparing patients with good versus intermediate Lung Immune Prognostic Index (LIPI).**

Panels (A–D) summarize subgroup meta-analyses assessing whether the prognostic association between LIPI and PFS varied according to (A) cancer type, (B) hazard ratios (HR) type (univariate vs. multivariate), (C) immune checkpoint inhibitor (ICI) treatment status, and (D) surgical treatment. Horizontal lines indicate 95% confidence intervals (CIs) for each subgroup, and diamonds represent pooled HRs.


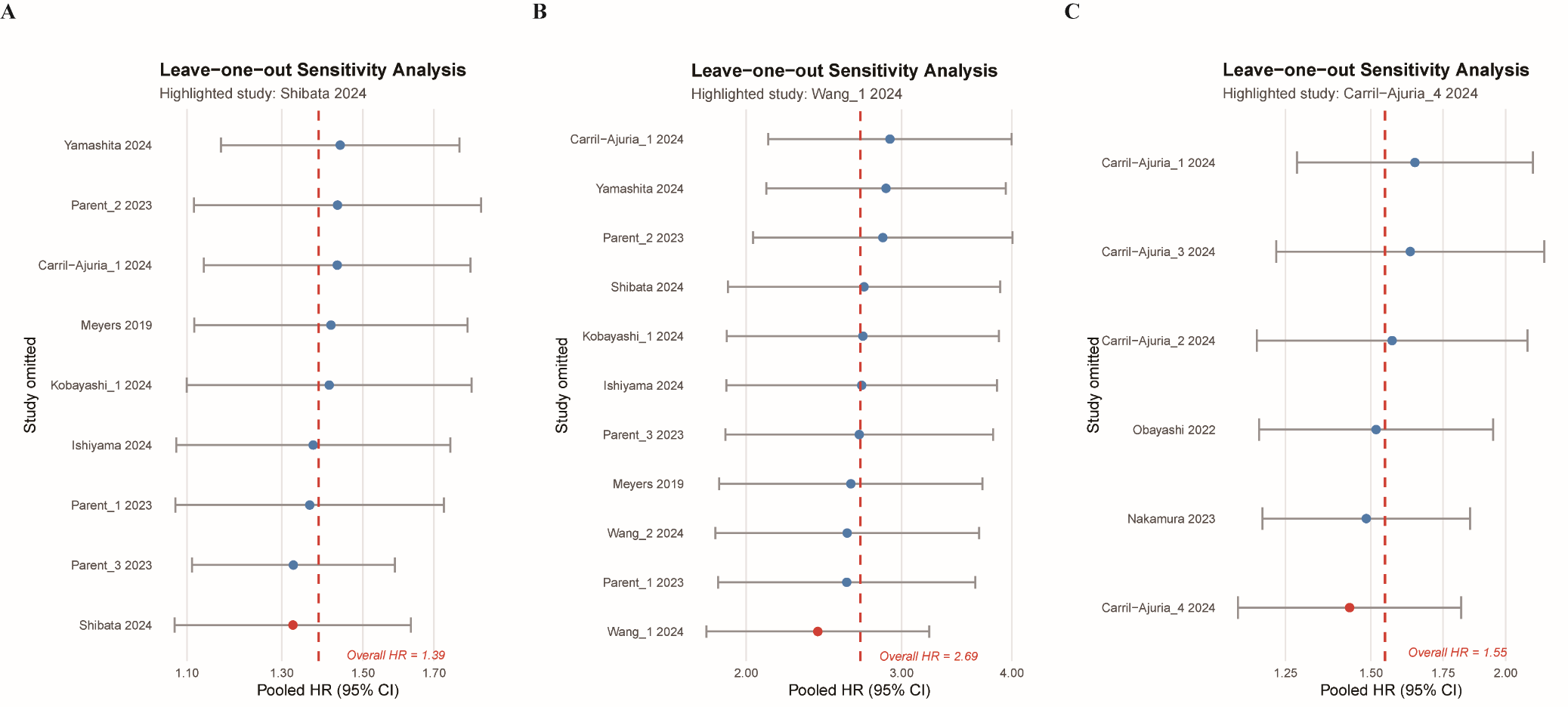


**Supplementary Figure 8. Leave-one-out sensitivity analysis assessing the robustness of pooled progress-free survival (PFS) estimates across different Lung Immune Prognostic Index (LIPI) comparisons.**

Panels (A–C) illustrate the results of sequentially omitting one study at a time to evaluate the influence of individual studies on the pooled hazard ratio (HR) for PFS in comparisons of (A) good vs. intermediate LIPI, (B) good vs. poor LIPI, and (C) good vs. intermediate or poor LIPI. The dashed horizontal line represents the overall pooled HR, while dots indicate recalculated HRs after each study’s exclusion. The minimal variation across panels demonstrates the stability and reliability of the pooled estimates.


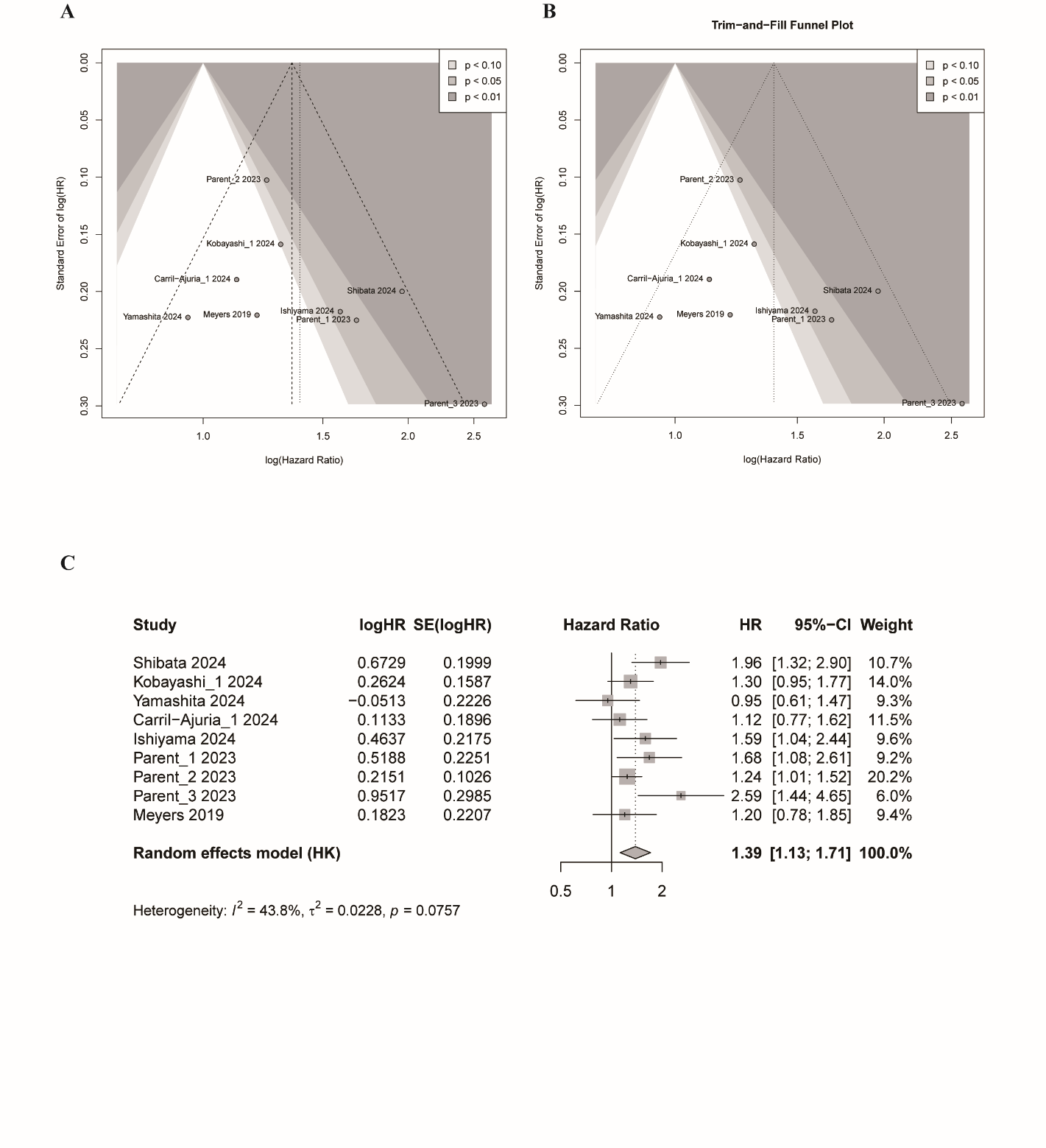


**Supplementary Figure 9. Publication bias evaluation for progress-free survival (PFS) comparing good versus intermediate Lung Immune Prognostic Index (LIPI).**

(A) Funnel plot of the included studies demonstrates visual assessment of potential publication bias. (B) Funnel plot adjusted by the trim-and-fill method, showing imputed studies (open circles) to estimate the potential effect of missing data. (C) Forest plot displaying the adjusted pooled hazard ratio (HR) and 95% confidence interval (CI) after applying the trim-and-fill method.


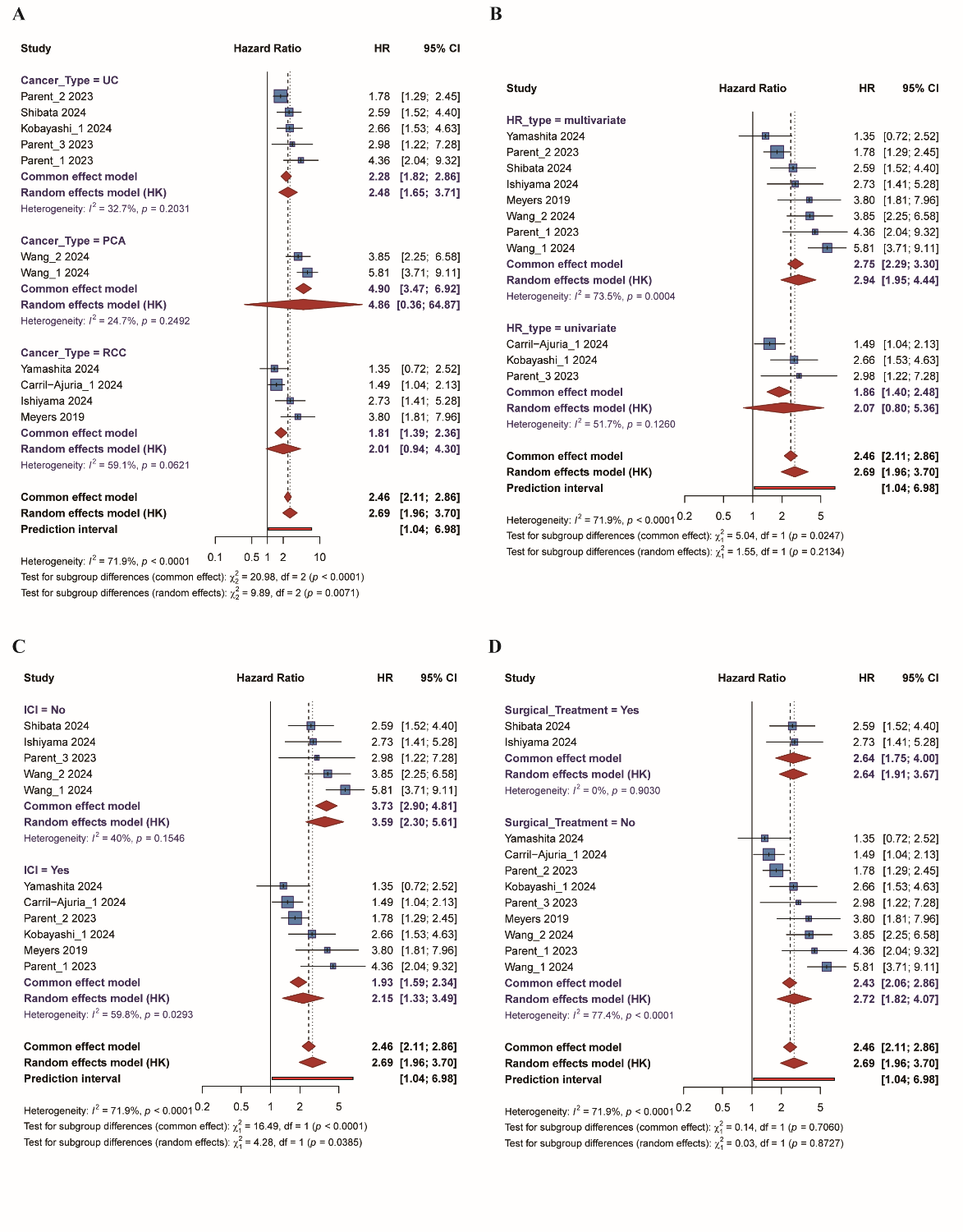


**Supplementary Figure 10. Subgroup analyses of progress-free survival (PFS) comparing patients with good versus poor Lung Immune Prognostic Index (LIPI).**

Panels (A–D) summarize subgroup meta-analyses assessing whether the prognostic association between LIPI and PFS varied according to (A) cancer type, (B) hazard ratios (HR) type (univariate vs. multivariate), (C) immune checkpoint inhibitor (ICI) treatment status, and (D) surgical treatment. Horizontal lines indicate 95% confidence intervals (CIs) for each subgroup, and diamonds represent pooled HRs.


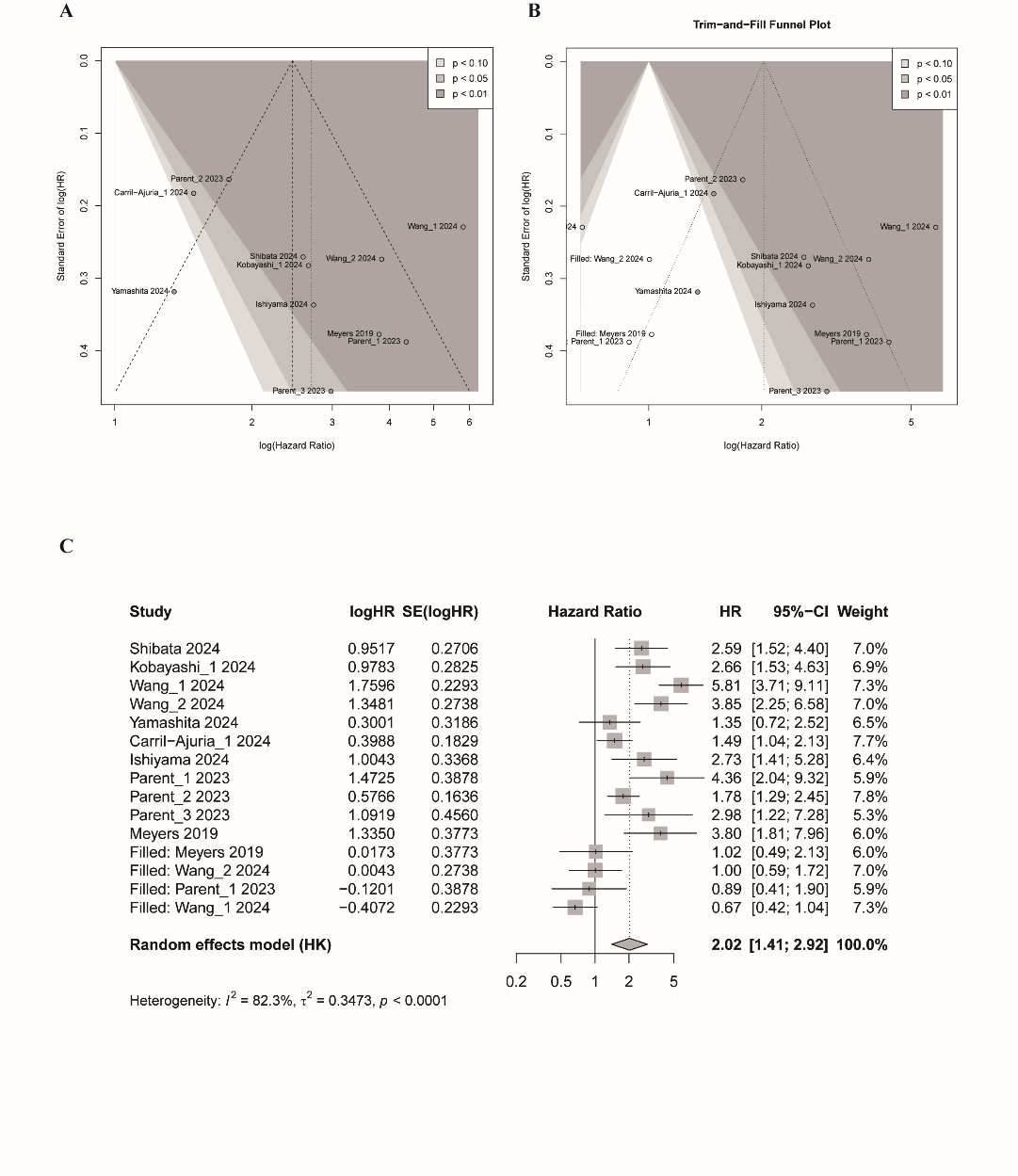


**Supplementary Figure 11. Publication bias evaluation for progress-free survival (PFS) comparing good versus poor Lung Immune Prognostic Index (LIPI).**

(A) Funnel plot of the included studies demonstrates visual assessment of potential publication bias. (B) Funnel plot adjusted by the trim-and-fill method, showing imputed studies (open circles) to estimate the potential effect of missing data. (C) Forest plot displaying the adjusted pooled hazard ratio (HR) and 95% confidence interval (CI) after applying the trim-and-fill method.


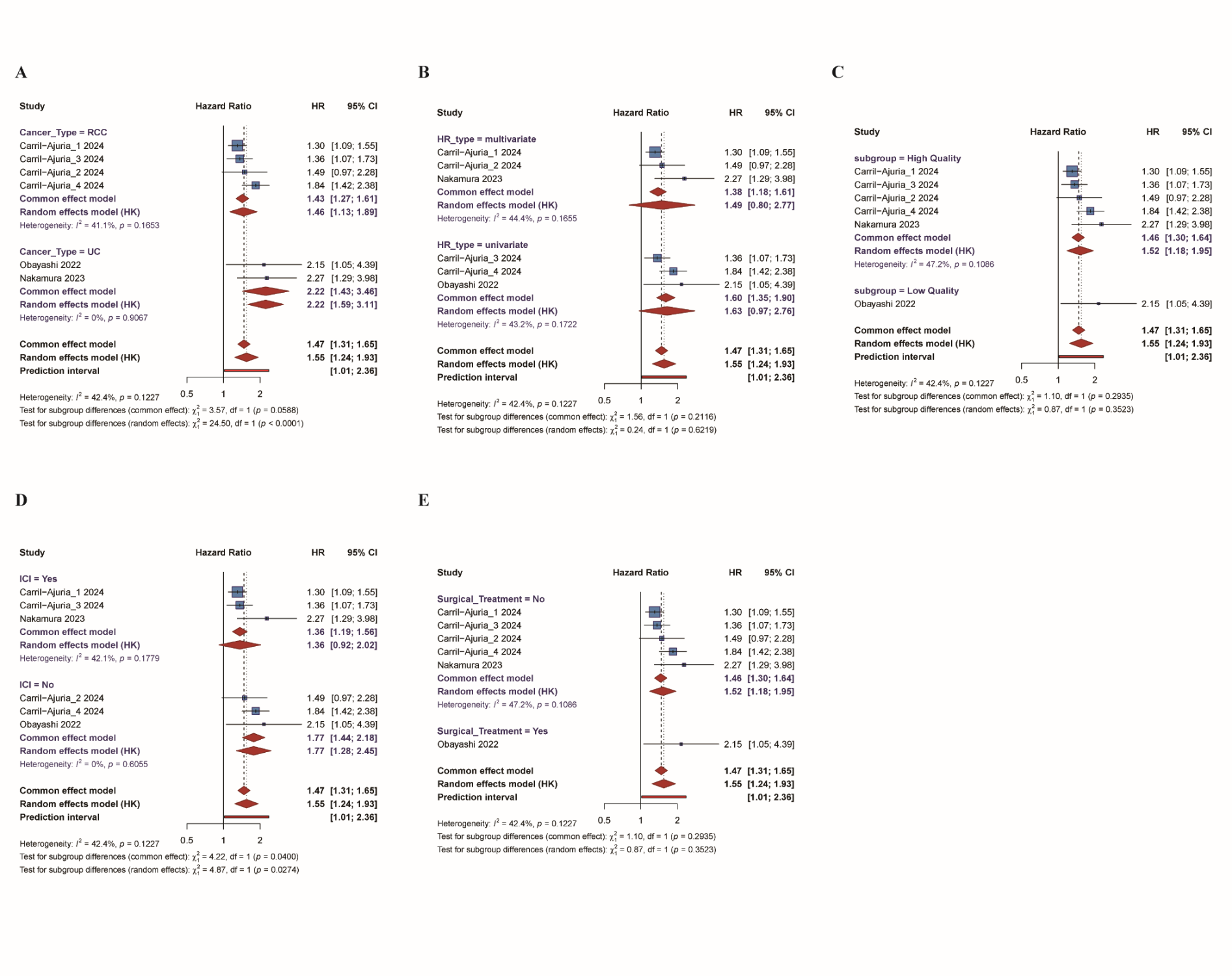


**Supplementary Figure 12. Subgroup analyses of progress-free survival (PFS) comparing patients with good versus intermediate or poor Lung Immune Prognostic Index (LIPI).**

Panels (A–D) summarize subgroup meta-analyses assessing whether the prognostic association between LIPI and PFS varied according to (A) cancer type, (B) hazard ratios (HR) type (univariate vs. multivariate), (C) immune checkpoint inhibitor (ICI) treatment status, and (D) surgical treatment. Horizontal lines indicate 95% confidence intervals (CIs) for each subgroup, and diamonds represent pooled HRs.


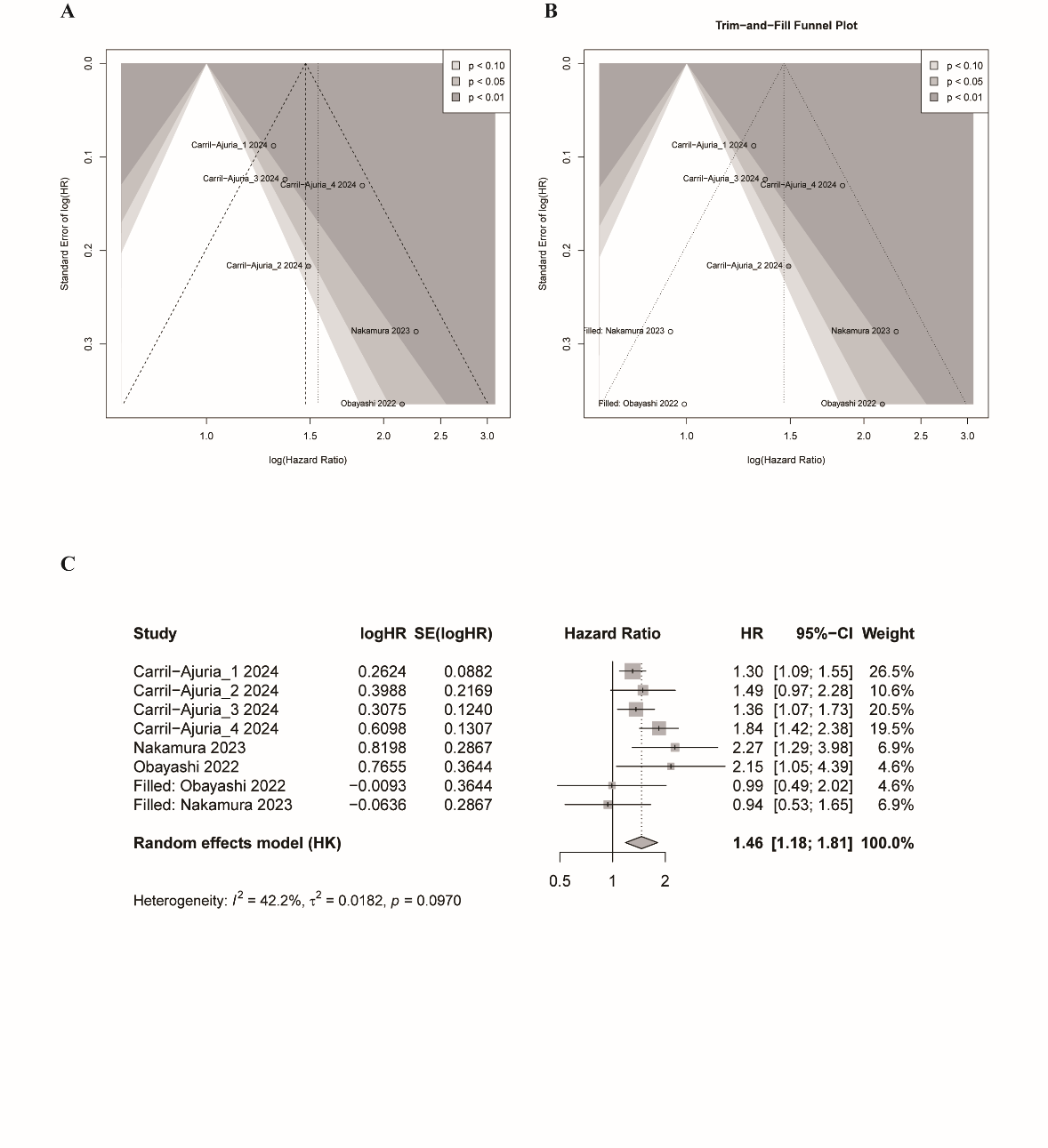


**Supplementary Figure 13. Publication bias evaluation for progress-free survival (PFS) comparing good versus intermediate or poor Lung Immune Prognostic Index (LIPI).**

(A) Funnel plot of the included studies demonstrates visual assessment of potential publication bias. (B) Funnel plot adjusted by the trim-and-fill method, showing imputed studies (open circles) to estimate the potential effect of missing data. (C) Forest plot displaying the adjusted pooled hazard ratio (HR) and 95% confidence interval (CI) after applying the trim-and-fill method.


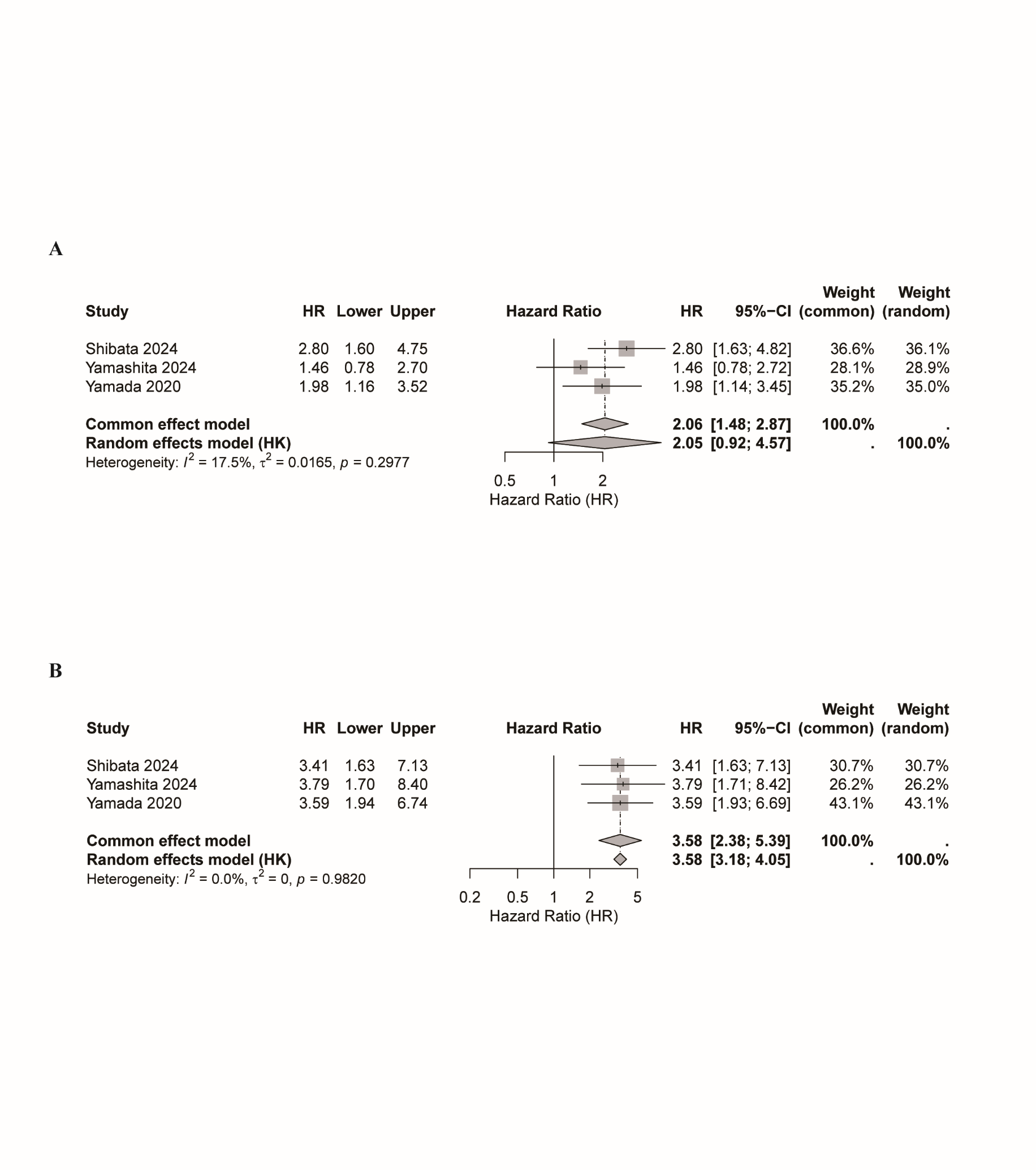


**Supplementary Figure 14. Forest plots of pooled cancer specific survival (CSS) according to Lung Immune Prognostic Index (LIPI) status.**

Meta-analytic comparisons were performed for (A) good vs. intermediate LIPI and (B) good vs. poor LIPI. Both Random-effects and common effect models were applied to estimate pooled hazard ratios (HRs) and 95% confidence intervals (CIs). The size of each square corresponds to the weight of the study, and the diamond represents the overall pooled estimate.


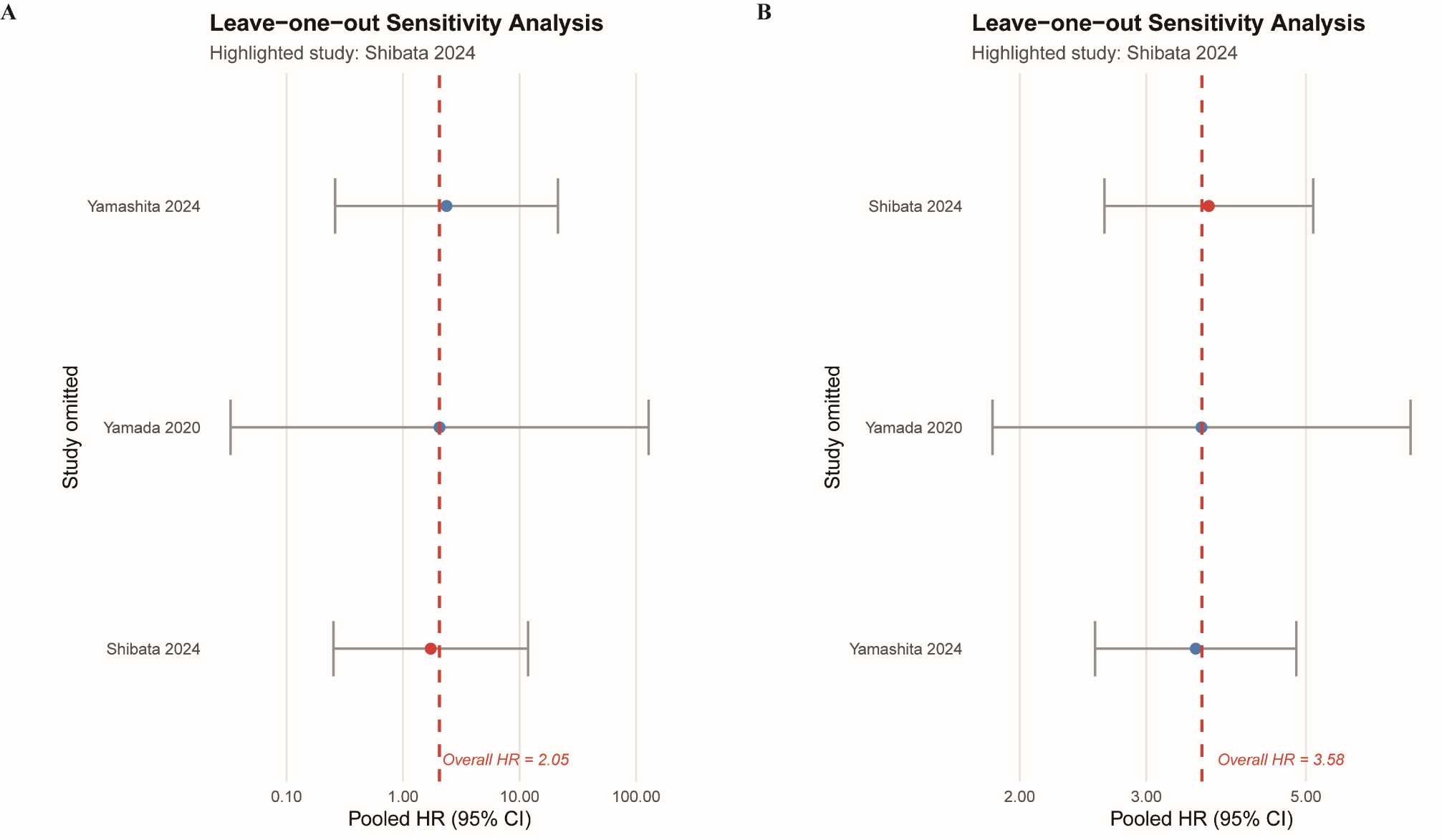


**Supplementary Figure 15. Leave-one-out sensitivity analysis assessing the robustness of pooled cancer specific survival (CSS) estimates across different Lung Immune Prognostic Index (LIPI) comparisons.**

Panels illustrate the results of sequentially omitting one study at a time to evaluate the influence of individual studies on the pooled hazard ratio (HR) for CSS in comparisons of (A) good vs. intermediate LIPI and (B) good vs. poor LIPI. The dashed horizontal line represents the overall pooled HR, while dots indicate recalculated HRs after each study’s exclusion. The minimal variation across panels demonstrates the stability and reliability of the pooled estimates.

**Supplementary Table 1. Methodological quality assessment of included studies using the Newcastle–Ottawa Scale.**

| **Author (Year)** | **Study design** | **Tumor type** | **Selection (0–4)** | **Comparability (0–2)** | **Outcome (0–3)** | **Total score (0–9)** | **Quality level*** |
| --- | --- | --- | --- | --- | --- | --- | --- |
| Hoshina (2025) | R | RCC | 4 | 0 | 1 | 5 | Moderate |
| Shibata (2024) | R | UC | 4 | 0 | 3 | 7 | High |
| Kobayashi (2024) | R | UC | 4 | 0 | 3 | 7 | High |
| Ou (2024) | R | UC | 4 | 0 | 1 | 5 | Moderate |
| Wang_1 (2024) | R | PCA | 4 | 1 | 3 | 8 | High |
| Wang_2 (2024) | R | PCA | 4 | 1 | 3 | 8 | High |
| Yamashita (2024) | R | RCC | 4 | 1 | 3 | 8 | High |
| Carril-Ajuria_1 (2024) | P | RCC | 4 | 1 | 3 | 8 | High |
| Carril-Ajuria_2 (2024) | P | RCC | 4 | 1 | 3 | 8 | High |
| Carril-Ajuria_3 (2024) | P | RCC | 4 | 1 | 3 | 8 | High |
| Carril-Ajuria_4 (2024) | P | RCC | 4 | 1 | 3 | 8 | High |
| Ishiyama (2024) | R | RCC | 4 | 2 | 3 | 9 | High |
| Nakamura (2023) | R | UC | 4 | 1 | 2 | 7 | High |
| Parent_1 (2023) | R | UC | 4 | 1 | 3 | 8 | High |
| Parent_2 (2023) | P | UC | 4 | 1 | 3 | 8 | High |
| Parent_3 (2023) | R | UC | 4 | 1 | 3 | 8 | High |
| Obayashi (2022) | R | UC | 4 | 0 | 2 | 6 | Moderate |
| Yamada (2020) | R | PCA | 4 | 0 | 3 | 7 | High |
| Meyers (2019) | R | RCC | 4 | 2 | 3 | 9 | High |
| R, retrospective study; P, prospective study; RCC, renal cell carcinoma; UC, urothelial carcinoma; PCA, prostate cancer.  *Scores ≥7 indicate high quality; scores of 5–6 indicate moderate quality; scores <5 indicate low quality. | | | | | | | |

**Supplementary Table 2. Meta-regression analysis of potential moderators affecting the pooled hazard ratios (HRs) for overall survival.**

| **Group** | **Moderator** | **β (95% CI)** | **p-value** | **R² (%)** |
| --- | --- | --- | --- | --- |
| Good vs. intermediate LIPI  (0 vs. 1) | Study quality  (NOS) | -0.16 (−0.34 to 0.02) | 0.072 | 100.0 |
|  | HR type | 0.11 (-0.17 to 0.39) | 0.391 | 0.0 |
|  | Cancer type  (RCC) | -0.25 (-0.84 to 0.35) | 0.369 | 0.0 |
|  | Cancer type  (UC) | -0.12 (-0.66 to 0.42) | 0.612 |  |
|  | ICI treatment | -0.24 (-0.46 to -0.02) | 0.037 | 100.0 |
|  | Surgical treatment | 0.29 (0.08 to 0.51) | 0.012 | 100.0 |
| Good vs. poor LIPI  (0 vs. 2) | Study quality  (NOS) | -0.12 (-0.46 to 0.23) | 0.478 | 0.0 |
|  | HR type | -0.06 (-0.57 to 0.44) | 0.783 | 0.0 |
|  | Cancer type  (RCC) | -0.69 (-1.15 to -0.22) | 0.009 | 100.0 |
|  | Cancer type  (UC) | -0.61 (-1.06 to -0.16) | 0.014 |  |
|  | ICI treatment | -0.28 (-0.74 to 0.18) | 0.204 | 26.4 |
|  | Surgical treatment | -0.20 (-0.98 to 0.59) | 0.586 | 0.0 |
| Good vs. intermediate/poor LIPI  (0 vs. 1 or 2) | Study quality  (NOS) | -0.11 (-0.56 to 0.35) | 0.576 | 0.6 |
|  | HR type | 0.09 (-1.06 to 1.24) | 0.849 | 0.0 |
|  | Cancer type | 0.58 (-0.14 to 1.30) | 0.091 | 32.6 |
|  | ICI treatment | 0.06 (-0.66 to 0.78) | 0.836 | 0.0 |
|  | Surgical treatment | 0.09 (-1.06 to 1.24) | 0.849 | 0.0 |

Random-effects meta-regression models were applied to evaluate the moderating effects of study-level covariates on the pooled HRs. R² indicates the proportion of between-study variance explained by each moderator. Bolded p-values represent statistically significant moderators (p < 0.05).

LIPI, lung immune prognostic index; NOS, Newcastle–Ottawa Scale; RCC, renal cell carcinoma; UC, urothelial carcinoma.

**Supplementary Table 3. Meta-regression analysis of potential moderators affecting the pooled hazard ratios (HRs) for progression-free survival.**

| **Group** | **Moderator** | **β (95% CI)** | **p-value** | **R² (%)** |
| --- | --- | --- | --- | --- |
| Good vs. intermediate LIPI  (0 vs. 1) | Study quality  (NOS) | -0.07 (−0.41 to 0.28) | 0664 | 0.0 |
|  | HR type | 0.03 (-0.48 to 0.53) | 0.901 | 0.0 |
|  | Cancer type | 0.26 (-0.15 to 0.67) | 0.181 | 0.0 |
|  | ICI treatment | -0.44 (-0.75 to -0.13) | **0.012** | 100.0 |
|  | Surgical treatment | 0.33 (-0.12 to 0.79) | 0.129 | 100.0 |
| Good vs. poor LIPI  (0 vs. 2) | Study quality  (NOS) | 0.09 (-0.48 to 0.66) | 0.723 | 0.0 |
|  | HR type | -0.32 (-1.05 to 0.40) | 0.339 | 2.7 |
|  | Cancer type  (RCC) | -0.90 (-1.62 to -0.17) | **0.022** | 59.3 |
|  | Cancer type  (UC) | -0.65 (-1.35 to -0.05) | 0.065 |  |
|  | ICI treatment | -0.52 (-1.09 to 0.06) | 0.072 | 44.6 |
|  | Surgical treatment | -0.02 (-0.91 to 0.86) | 0.958 | 0.0 |
| Good vs. intermediate/poor LIPI  (0 vs. 1 or 2) | Study quality  (NOS) | -0.26 (-0.68 to 0.16) | 0.163 | 35.6 |
|  | HR type | 0.10 (-0.43 to 0.62) | 0.634 | 0.0 |
|  | Cancer type | 0.42 (-0.17 to 1.01) | 0.117 | 40.6 |
|  | ICI treatment | -0.26 (-0.64 to 0.11) | 0.124 | 100.0 |
|  | Surgical treatment | 0.35 (-0.76 to 1.45) | 0.430 | 5.9 |

Random-effects meta-regression models were applied to evaluate the moderating effects of study-level covariates on the pooled HRs. R² indicates the proportion of between-study variance explained by each moderator. Bolded p-values represent statistically significant moderators (p < 0.05).

LIPI, lung immune prognostic index; NOS, Newcastle–Ottawa Scale; RCC, renal cell carcinoma; UC, urothelial carcinoma.
